# Resolving Mixed Intermediate Phases in Methylammonium-Free Sn–Pb Alloyed Perovskites for High-Performance Solar Cells

**DOI:** 10.1007/s40820-022-00918-1

**Published:** 2022-08-16

**Authors:** Zhanfei Zhang, Jianghu Liang, Jianli Wang, Yiting Zheng, Xueyun Wu, Congcong Tian, Anxin Sun, Zhenhua Chen, Chun-Chao Chen

**Affiliations:** 1 School of Materials Science and Engineering, Shanghai Jiao Tong University, Shanghai, 20024 People’s Republic of China; 2 Shanghai Synchrotron Radiation Facility (SSRF), Shanghai Advanced Research Institute, Chinese Academy of Sciences, Shanghai, 201800 People’s Republic of China

**Keywords:** Intermediate phase, Homogeneous nucleation process, MA-free tin–lead alloyed perovskite, Light and thermal stability, Tandem device

## Abstract

The complete elimination of methylammonium (MA) cations in Sn–Pb composites can extend their light and thermal stabilities. Unfortunately, MA-free Sn–Pb alloyed perovskite thin films suffer from wrinkled surfaces and poor crystallization, due to the coexistence of mixed intermediate phases. Here, we report an additive strategy for finely regulating the impurities in the intermediate phase of Cs_0.25_FA_0.75_Pb_0.6_Sn_0.4_I_3_ and, thereby, obtaining high-performance solar cells. We introduced *d*-homoserine lactone hydrochloride (D-HLH) to form hydrogen bonds and strong Pb–O/Sn–O bonds with perovskite precursors, thereby weakening the incomplete complexation effect between polar aprotic solvents (e.g., DMSO) and organic (FAI) or inorganic (CsI, PbI_2_, and SnI_2_) components, and balancing their nucleation processes. This treatment completely transformed mixed intermediate phases into pure preformed perovskite nuclei prior to thermal annealing. Besides, this D-HLH substantially inhibited the oxidation of Sn^2+^ species. This strategy generated a record efficiency of 21.61%, with a *V*_oc_ of 0.88 V for an MA-free Sn–Pb device, and an efficiency of 23.82% for its tandem device. The unencapsulated devices displayed impressive thermal stability at 85 °C for 300 h and much improved continuous operation stability at MPP for 120 h.

**Supplementary Information:**

The online version contains supplementary material available at 10.1007/s40820-022-00918-1.

## Highlights

- The first study dedicated to solving the problem of mixed intermediate phases in methylammonium (MA)-free (FA-Cs) Sn–Pb alloyed perovskite systems, new performance records of 21.61% and 23.82% realized for its single-junction and tandem devices.
- d-Homoserine lactone hydrochloride (D-HLH), employed as an additive, accelerates the transition from mixed intermediate phases into preformed perovskite nuclei.
- The presence of D-HLH eliminated the negative effect of mixed intermediate phases and regulated the crystallization kinetics.

**Supplementary Information:**

The online version contains supplementary material available at 10.1007/s40820-022-00918-1.

## Graphical abstract

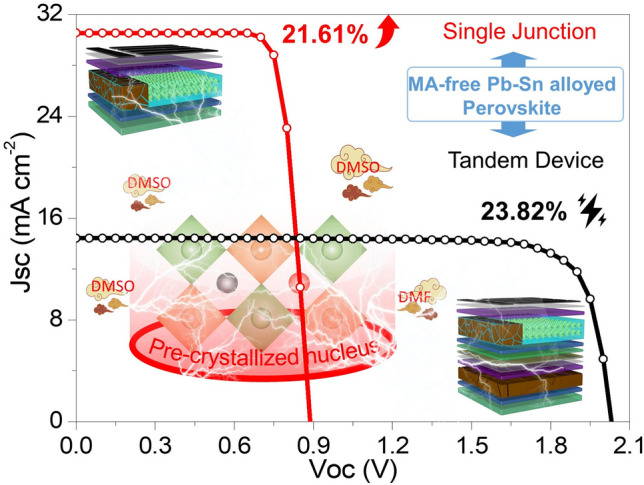

## Introduction

The optimal certified power conversion efficiency (PCE) for a lead (Pb) halide perovskite solar cell (PSC) has now reached over 25% [1]. Similarly, the highest reported PCE of a mixed tin–lead (Sn–Pb) PSC is 23.6%, realized after treatment with ethylenediammonium (EDA) and glycinium (GlyH) as surface passivators [2]. Despite this notable success, current Sn–Pb alloyed PSCs still display stubborn shortcomings in terms of stability. First of all, Sn-containing perovskites face serious intrinsic chemical instability because Sn atoms can be oxidized from the 2 + to the 4 + state, leading to high trap densities and even complete breakdown of the perovskite absorber [3, 4]. To address this limitation, many efforts (e.g., purification of the tin source; use of antioxidant additives; device engineering strategies) have been made to decrease Sn^4+^ densities [5–9]. Unfortunately, the resulting Sn–Pb alloyed perovskites still undergo rapid decay during device performance upon exposure to elevated temperatures and testing at the maximum power point (MPP) under continuous illumination [10–14]. The root cause of this thermal and light instability is the large content (> 30%) of volatile methylammonium (MA) cations in these highly efficient (PCE > 20%) Sn–Pb alloyed PSCs [15–17]. To realize the full elimination of MA, a cesium-formamidinium (Cs-FA)–based Sn–Pb alloyed perovskite material has been prepared, displaying improved light and thermal stabilities [18–21]. Nevertheless, the reported performances of MA-free Sn–Pb alloyed PSCs remain far behind those of MA-containing Sn–Pb devices, because of low open-circuit voltages (*V*_oc_) and fill factors (FFs) (Fig. S1, Table S1). Therefore, it will be necessary to determine the root cause of the inferior performance of MA-free Sn–Pb alloyed PSCs to further improve their stability.

The crystallization process of a Sn–Pb alloyed perovskite film is quite different from that of a single-phase perovskite, because both Sn- and Pb-based perovskite phases coexist and nucleate from the mixed precursor solution. The most common method for preparing Sn–Pb alloyed perovskite films is through a one-step antisolvent process, where the excess solvent contained in the films is extracted by the dripping of an antisolvent to form a transitional intermediate phase [22]. The intermediate phase that provides the nucleation sites then promotes crystallization of the film during annealing [23]. Nevertheless, the presence of such intermediate phases in different perovskite composites is not always well understood or controlled. Recently, McGehee et al. observed a problematic mixed intermediate phase in an FA_0.75_Cs_0.25_Sn_0.5_Pb_0.5_I_3_ film; their antisolvent-treated MA-free Sn–Pb alloyed perovskite film featured surface wrinkles and suffered from poor crystal quality after annealing, but no explanation related to the intermediate phase was given [24]. Therefore, more attention should be paid to solving the problems arising from mixed intermediate phases in MA-free Sn–Pb alloyed perovskites to realize high-performance devices.

To form the intermediate phases, polar aprotic solvents [e.g., dimethylformamide (DMF) and dimethyl sulfoxide (DMSO)] are commonly employed because of their strong coordination with the perovskite precursors [25–27]. Specifically, DMSO has a higher donor number than DMF, resulting in a shorter bond length when coordinating with the perovskite components (the so-called complexation effect) [28, 29] The presence of PbI_2_–DMSO complexes can retard crystallization of the perovskite through the formation of a stable MAI–DMSO–PbI_2_ intermediate phase, which is then converted to a homogeneous three-dimensional (3D) perovskite phase upon annealing [30]. Nevertheless, full replacement of MAI with FAI (formamidinium iodide) can lead to a less-effective intermediate phase [31]. For example, Li et al. reported that the charge densities of DMSO and FA^+^ were isolated, and that a lower degree of electron transfer occurred between DMSO and FA^+^, thereby weakening the stability of the FAI–DMSO–PbI_2_ intermediate phase, resulting in a rapid transition of the non-perovskite phase (δ-FAPbI_3_) and uncontrollable phase separation of PbI_2_ [32, 33]. To solve this problem, CsI is always incorporated to minimize halide (iodide, I^–^) phase segregation [34, 35]. Nevertheless, Luther et al. demonstrated that CsI can complex strongly with DMSO; the resulting CsI–DMSO adduct had greater control over the nucleation of the perovskite than did the PbI_2_–DMSO adduct [36]. Moreover, for Sn^2+^-containing systems, Ning et al. reported that the Lewis acidity of Sn^2+^ is higher than that of Pb^2+^; the resulting strong interaction between Sn^2+^ and I^–^ weakened the coordination of PbI_2_ with DMSO [37]. Therefore, in Cs-FA-containing Sn–Pb alloyed perovskite precursors, DMSO alone cannot balance the interactions among the components (Sn^2+^, FA^+^, and Cs^+^), resulting in mixed intermediate phases and uncontrolled nucleation processes.

Because the intermediate phase is not always beneficial to the fabrication of high-quality perovskite films, some approaches have been developed to minimize the presence of intermediate phases for Pb-based perovskites. For example, Han et al. introduced ethyl acetate as an antisolvent to induce instant crystallization of perovskite films without the presence of an intermediate phase [38]. Ding et al. adopted *N*-methyl-2-pyrrolidone (NMP) and dimethylacetamide (DMAc) as co-solvents to decrease the transition window of intermediate phase [39]. Furthermore, Lee et al. used trimethyl phosphate (TMP), having a low donor number, as a replacement to DMSO, thereby realizing an intermediate phase–free process for preparing high-performance PSC [40]. Therefore, accelerating the transition from the intermediate phase into the perovskite phase or directly forming the perovskite phase without inducing an intermediate phase can be a potential solution to eliminate the negative effect of mixed intermediate phases in MA-free Sn–Pb alloyed perovskites.

In this study, we developed a new additive strategy to finely regulate the intermediate phase of Cs_0.25_FA_0.75_Pb_0.6_Sn_0.4_I_3_ for high-performance MA-free Sn–Pb alloyed PSCs. Considering that the cyclic lactone molecules, such as *γ*-butyrolactone (GBL), are used as solvent-additive to generate complexation with the precursor components to optimize the perovskite crystallization process, the amine group (− NH_2_) has been shown to be effective in passivating perovskite crystal defects [18, 41–43]. We introduced d-homoserine lactone hydrochloride (D-HLH) that meets all these requirements to achieve specific bonding interactions with the various perovskite components. D-HLH can form hydrogen bonds [–O–C(= O)**···**H–N and –NH**···**I^–^] and strong Pb–O and Sn–O bonds with many of the perovskite precursors, thereby weakening the incomplete complexation effect between polar aprotic solvents (e.g., DMSO) and organic (FAI) or inorganic (CsI, PbI_2_, and SnI_2_) components, and unifying the nucleation process of each component. These phenomena promote the complete transformation of the mixed intermediate phases into pure preformed perovskite nuclei, resulting in a homogeneous nucleation process for effective regulation of the crystallization kinetics. In addition, these specific bonding interactions substantially inhibit the absorption and oxidation processes caused by ambient oxygen, greatly contributing to film stability. As a result, our MA-free Sn–Pb alloyed PSC operated with a lowest *V*_oc_ loss of 0.36 V, a high FF of 80.36%, and a recorded efficiency of 21.61%. Furthermore, was fabricated a corresponding tandem device that pushed the PCE up to 23.82%. The unencapsulated device also displayed impressive thermal stability, retaining 95.1% of the initial PCE after heating at 85 °C for 300 h, and displayed much improved continuous operation stability, retaining 98.3% of its initial PCE after 120 h with MPP tracking under 1-sun illumination. Thus, overcoming an intrinsic nucleation problem that restricts the crystallization process, by employing a new additive engineering strategy, can realize high-efficiency, highly stable MA-free Sn–Pb alloyed perovskite systems, with great enhancements in the performance of corresponding tandem devices.

## Experimental

### Materials

Cesium iodide (CsI, > 99.9%), formamidinium iodide (FAI, > 99.5%), methylammonium iodide (MAI, > 99.5%), lead(II) iodide (PbI_2_, > 99.99%), lead(II) bromide (PbBr_2_, > 99.99%), bathocuproine (BCP), and [6, 6]-phenyl-C_61_-butyric acid methyl ester (PCBM) were purchased from Xi’an Polymer Light Technology. Tin(II) iodide (SnI_2_, 99.99%), tin(II) fluoride (SnF_2_, 99%), nickel(II) nitrate hexahydrate [Ni(NO_3_)_2_**·**6H_2_O], 2-methoxyethanol, acetylacetone, ammonia, *N*,*N*-dimethylformamide (DMF, 99.8%), dimethyl sulfoxide (DMSO, 99.8%), isopropanol (IPA), chlorobenzene (CB), γ-butyrolactone (GBL), and anisole were obtained from Sigma-Aldrich. d-Homoserine lactone hydrochloride (D-HLH) was purchased from Macklin. Poly(3,4-ethylenedioxythiophene):polystyrenesulfonate (PEDOT:PSS, Clevios™ PVP AI 4083) was purchased from Heraues.

### Solution Preparation

A 1.8 M solution of the low-band-gap MA-free Sn–Pb alloyed perovskite Cs_0.25_FA_0.75_Pb_0.6_Sn_0.4_I_3_ precursor was prepared by dissolving 0.117 g of CsI, 0.232 g of FAI, 0.498 g of PbI_2_, 0.268 g of SnI_2_, 0.011 g of SnF_2_, and various amounts of D-HLH in a mixture of DMSO and DMF (1:3, v/v). A 1.2 M solution of the wide-band-gap Cs_0.05_FA_0.8_MA_0.15_Pb(I_0.6_Br_0.4_)_3_ precursor was prepared by dissolving 0.165 g of FAI, 0.016 g of CsI, 0.029 g of MAI, 0.221 g of PbI_2_, and 0.264 g of PbBr_2_ in a mixture of DMF, DMSO, and GBL (6:2:2, v/v/v). The perovskite solutions were passed through polytetrafluoroethylene (PTFE) filters (0.22 μm) prior to use. The nickel(II) nitrate solution for preparing the NiO_x_ layer was obtained by dissolving 290.8 mg of Ni(NO_3_)_2_**·**6H_2_O in 5 mL of 2-methoxyethanol. The 200 mL of acetylacetone were then added sequentially to this solution. Solutions of 20 mg mL^–1^ PCBM in CB and 5 mg mL^–1^ BCP in IPA were prepared to form the electron transport material and hole barrier layer, respectively, of the devices.

### MA-free Sn–Pb Alloyed Low-band Gap Device Fabrication

Glass/ITO substrates (2.0 × 2.0 cm^2^) were washed sequentially with deionized water, absolute ethanol, acetone, and isopropanol in an ultrasonic bath for 20 min. The ITO substrates were then further cleaned through UV-ozone treatment for 15 min. After cooling to room temperature, the PEDOT:PSS solution was spin-coated onto the ITO substrates (4000 rpm, 30 s), which were then annealed (150 °C, 10 min) in ambient air. The PEDOT:PSS-coated substrates were placed in a N_2_-filled glove box. The Cs_0.25_FA_0.75_Pb_0.6_Sn_0.4_I_3_ perovskite films were deposited by spin-coating the precursor solution onto the PEDOT:PSS film (4000 rpm, 30 s). Anisole (200 μL) was dropped onto the spinning substrate 25 s after the onset of spinning. The perovskite films were annealed at 100 °C for 7 min. Next, PCBM (1000 rpm, 30 s) and BCP (5000 rpm, 30 s) were deposited by spin-coating. Finally, Ag (100 nm) was deposited as the back electrode through a shallow mask by thermal evaporation under a pressure of 9 × 10^–5^ Pa. The total device active area of single-junction cell was 0.18 cm^2^, and the mask aperture area was 0.1 cm^2^. All measurements were made with unencapsulated devices and performed exposed to ambient air (RH = 30 ± 5%) or in a N_2_-filled glove box at room temperature.

### Monolithic All-perovskite Tandem Device Fabrication

The glass/ITO substrate was washed successively with absolute ethanol, detergent, deionized water, acetone, and isopropanol in an ultrasonic bath for 20 min. Prior to using the substrate, it was treated with UV-ozone for 15 min. After cooling to room temperature, the NiO_x_ hole transport layer was spin-coated onto the ITO substrate using the combustion method. The nickel(II) nitrate solution was spin-coated (4000 rpm, 30 s) onto the ITO, which was then annealed in ambient air at 250 °C for 30 min before cooling to room temperature. The wide-band-gap Cs_0.05_FA_0.8_MA_0.15_Pb(I_0.6_Br_0.4_)_3_ precursor solution (80 μL) was dropped onto the NiO_x_-coated ITO substrate, which was spin-coated at 4000 rpm for 40 s. Then, 8 s before the end of the program, CB (100 μL) was slowly dropped onto the film as an antisolvent. After spin-coating, the sample was annealed at 115 °C for 20 min. Next, PCBM was spin-coated (2000 rpm, 30 s). The substrates were transferred to an atom layer deposition (ALD) system (NCE-200R, NANOFRONT IER MEE) and a 30-nm SnO_2_ film was deposited at low temperature (100 °C) using ((CH_3_)_2_ N)_4_Sn and deionized water as Sn and O sources, respectively. After ALD deposition, the substrates were returned to the thermal evaporation system to deposit the Au clusters layer (ca. 1 nm) on the ALD-SnO_2_. PEDOT:PSS layers were spin-coated on top of the ITO layers and annealed in air at 120 °C for 20 min. After the substrates had cooled, they were immediately transferred to the N_2_-filled glove box for deposition of low-band-gap perovskite films, using procedures identical to those described for the single-junction device. The bandgap values of wide-band-gap and low-band-gap perovskite films are 1.77 and 1.24 eV, respectively [18, 43]. The total device active area of tandem device was 0.18 cm^2^, and the mask aperture area was 0.1 cm^2^.

### Characterization

The *J*–*V* characteristics of the as-prepared devices were determined by a Keithley 2400 source meter under a simulated AM 1.5G spectrum at 100 mW cm^–2^ [Abet Technologies Sun 2000 solar simulator, calibrated with a standard VLSI Si reference solar cell (SRC-1000-TC-K-QZ)], with reverse scanning ranging from + 1.0 to –0.2 V with an interval of 50 mV s^–1^ and forward scanning ranging from –0.2 to + 1.0 V with the same steps. External quantum efficiency (EQE) spectra were recorded using a QTEST HIFINITY 5 EQE system (light intensity was calibrated with Si detectors) in ambient air. X-ray diffraction (XRD) patterns were recorded using a Bruker D8 Advance X-ray diffractometer under Cu K_α_ radiation over a 4–50° scan range with a step size of 0.02°s^–1^. Field emission scanning electron microscopy (FESEM) images were acquired using a JSM-7800F FESEM instrument. Field emission transmission electron microscopy (FETEM) images were obtained using a Talos F200X G2 instrument. Kelvin probe force microscopy (KPFM) and atom force microscopy (AFM) were performed using a Bruker Bio-FastScan AFM instrument operated in tapping mode. Steady-state photoluminescence (PL) and time-resolved photoluminescence (TRPL) spectra were recorded using an FLS 1000 photoluminescence spectrometer with light incident from the perovskite film side; the excitation wavelength was 405 nm. Dynamic light scattering (DLS) was performed using a Zetasizer Nano S nanoparticle size analyzer. X-ray photoelectron spectra (XPS) were recorded using a Thermo Scientific K-Alpha X-ray photoelectron spectrometer and calibrated to the C 1* s* binding energy; curve fitting was performed using Thermo Avantage software. Fourier transform infrared (FTIR) spectra were recorded using a Thermo-Nicolet iS5 instrument. Time-of-flight secondary ion mass spectrometry (ToF–SIMS) was performed using a Germany ION-TOF ToF-SIMS 5–100 system. Electrochemical impedance spectroscopy (EIS) was performed using a Chenhua CHI660E electrochemical workstation under dark conditions. Grazing incidence wide-angle X-ray scattering (GIWAXS) was performed at beamline BL16B1 at the Shanghai Synchrotron Radiation Facility (SSRF); the incidence angle of the X-ray beam was 0.3°, the normal incidence of the SSRF 16B was approximately 400 µm, and the grazing incidence spot could reach the centimeter-level [grazing incidence angle: 0.3°; the spot along the optical path could be elongated to 400/sin°(0.3°) = 7.6 cm; spot area: ca. 400 μm × 7.6 cm].

## Results and Discussion

### Crystal Growth Process

**Fig. 1:**
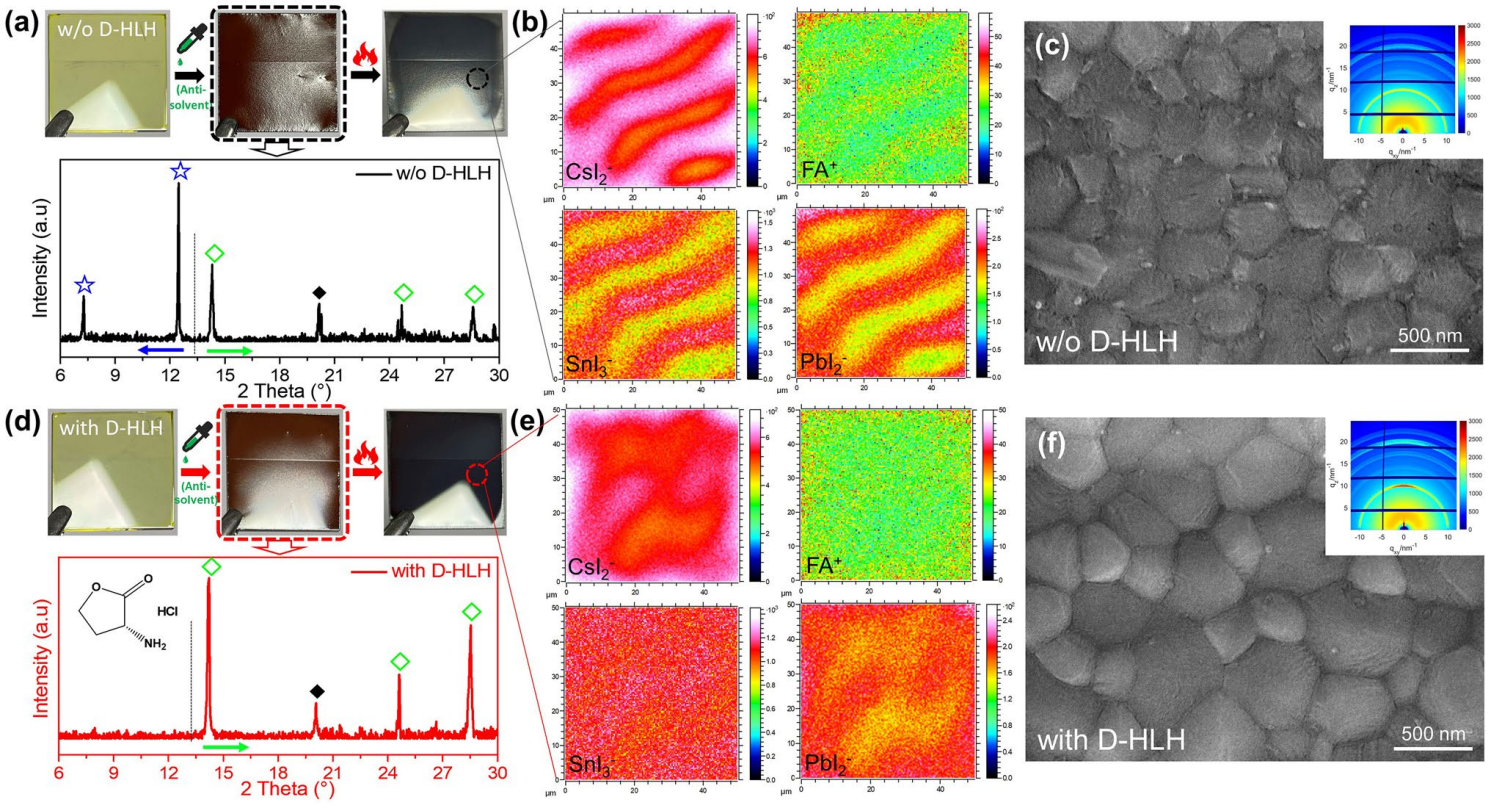
**a, d** Photographs taken during the perovskite film fabrication process and XRD patterns of the intermediate phase films prepared **a** without or **d** with D-HLH treatment (◆ represents ITO). **b, e** ToF–SIMS total depth profiles of the perovskites prepared **b** without and **e** with D-HLH. **c, f** SEM images of perovskites prepared **c** without and **f** with D-HLH; insets: GIWAXS images of the corresponding films

We used a one-step deposition technique to fabricate the MA-free Sn–Pb alloyed perovskite (Cs_0.25_FA_0.75_Pb_0.6_Sn_0.4_I_3_) films. Figure 1a displays real-time photographs of the perovskite film (control sample) taken during its fabrication process. After treatment with the antisolvent, the intermediate-phase film was dark-brown and had an unusually rough and uneven surface. Moreover, discontinuous wrinkles remained in the final films after annealing. Next, we applied XRD to determine the crystallographic structure of the intermediate phase in the control sample. Figure 1a reveals the coexistence of the intermediate phase (diffraction peaks at 7.2° and 12.4°) and the perovskite phase (diffraction peaks at 14.2°, 24.6°, and 28.5°) [44, 45]. We denote this situation, of both intermediate and perovskite phases prior to thermal annealing, as a “mixed intermediate phase.” We then used time-of-flight secondary ion mass spectrometry (ToF–SIMS) total depth profiles to evaluate the elemental distributions in the annealed film (Fig. 1b). Various perovskite components, including CsI_2_^–^, SnI_3_^–^, PbI_2_^–^, and FA^+^, existed in aggregated distributions in the vertical direction (top) of the final film. In other words, the mixed intermediate phase triggered the formation of heterogeneous nucleation sites, resulting in aggregated crystallization of the various components. As a result, we obtained a poorly crystallized perovskite film having a wrinkled surface. Notably, this paper is the first to report the visualization of a wrinkled MA-free Sn–Pb alloyed perovskite film from quantitative elemental distribution analysis. We applied both SEM and grazing incidence wide-angle X-ray scattering (GIWAXS) measurements to further characterize the final film quality of our control sample (Fig. 1c). As expected, SEM imaging revealed wrinkles spread over the surface of small grains. Although the GIWAXS image contained a strong diffraction ring at a value of *q*_*xy*_ of 10 nm^–1^, corresponding to the (110) perovskite crystal plane, there was no specific diffraction intensity, especially at an azimuthal angle of 90°, indicating the poor crystalline quality of the annealed film of the control sample. In previous studies we found that the nucleation and crystallization of the Sn component in Sn–Pb alloyed perovskites can be much faster than those of the Pb component, starting from the intermediate phase [22]. In other words, the major problem with a mixed intermediate phase having multiple components (e.g., intermediate and perovskite phases) is that the nucleation and crystallization of each component will proceed at different rates, resulting in phase segregation, distorted crystal structures, and poorly crystallized film morphologies [46]. Therefore, to achieve effective regulation of the crystallization kinetics, the first step would be to unify the nucleation rates of the various intermediate phases to ensure homogeneous nucleation sites for subsequent crystallization processes.

The intermediate phase is formed by coordinating perovskite precursors with solvents and/or additives prior to formation of the final film. Nevertheless, the nucleation processes for the highly reactive ions Cs^+^ and Sn^2+^ are much different than those for FA^+^ and Pb^2+^. As a result, DMSO-containing intermediate adducts failed at unifying the nucleation processes in our system. Thus, we developed an approach to accelerate the transition from a mixed intermediate phase into preformed perovskite nuclei and eliminate the negative effect of the intermediate adducts on the crystal growth process. Accordingly, we introduced D-HLH, which contains polar ester [–O–C(= O)–] and amino [–NH_2_] functional groups and has excellent solubility in DMF/DMSO, into the perovskite precursor solution. To examine the effect of D-HLH, we characterized the D-HLH-treated Cs_0.25_FA_0.75_Pb_0.6_Sn_0.4_I_3_ film. The real-time photographs in Fig. 1d reveal that the dark-brown intermediate-phase film was smooth and uniform, whereas the annealed film treated with D-HLH had a shiny surface and reflected mirror-like images. The XRD pattern (Fig. 1d) of the D-HLH-treated intermediate phase contained peaks only at 14.2°, 24.6°, and 28.5°, corresponding to a pure perovskite phase; no other redundant peaks were present. Furthermore, the TOF–SIMS total depth profile (Fig. 1e) revealed that the perovskite components were distributed uniformly throughout the whole annealed film, indicating much improved crystallization. The SEM and GIWAXS patterns in Fig. 1f indicate that D-HLH treatment led to a neat grain surface having much larger grains, as well as a large increase in intensity at the azimuthal angle of 90° in the (110) diffraction ring, consistent with oriented crystal growth and improved crystallinity. In sum, the D-HLH treatment prevented the formation of a mixed intermediate phase by transforming the various intermediate adducts into pure perovskite nuclei immediately after antisolvent dripping. With only pure perovskite nuclei and no intermediate phase in the film, the crystallization process was better regulated during subsequent thermal annealing, resulting in the improved crystal quality of the Cs_0.25_FA_0.75_Pb_0.6_Sn_0.4_I_3_ film.

### Accelerating the Formation of Preformed Perovskite Nuclei

**Fig. 2:**
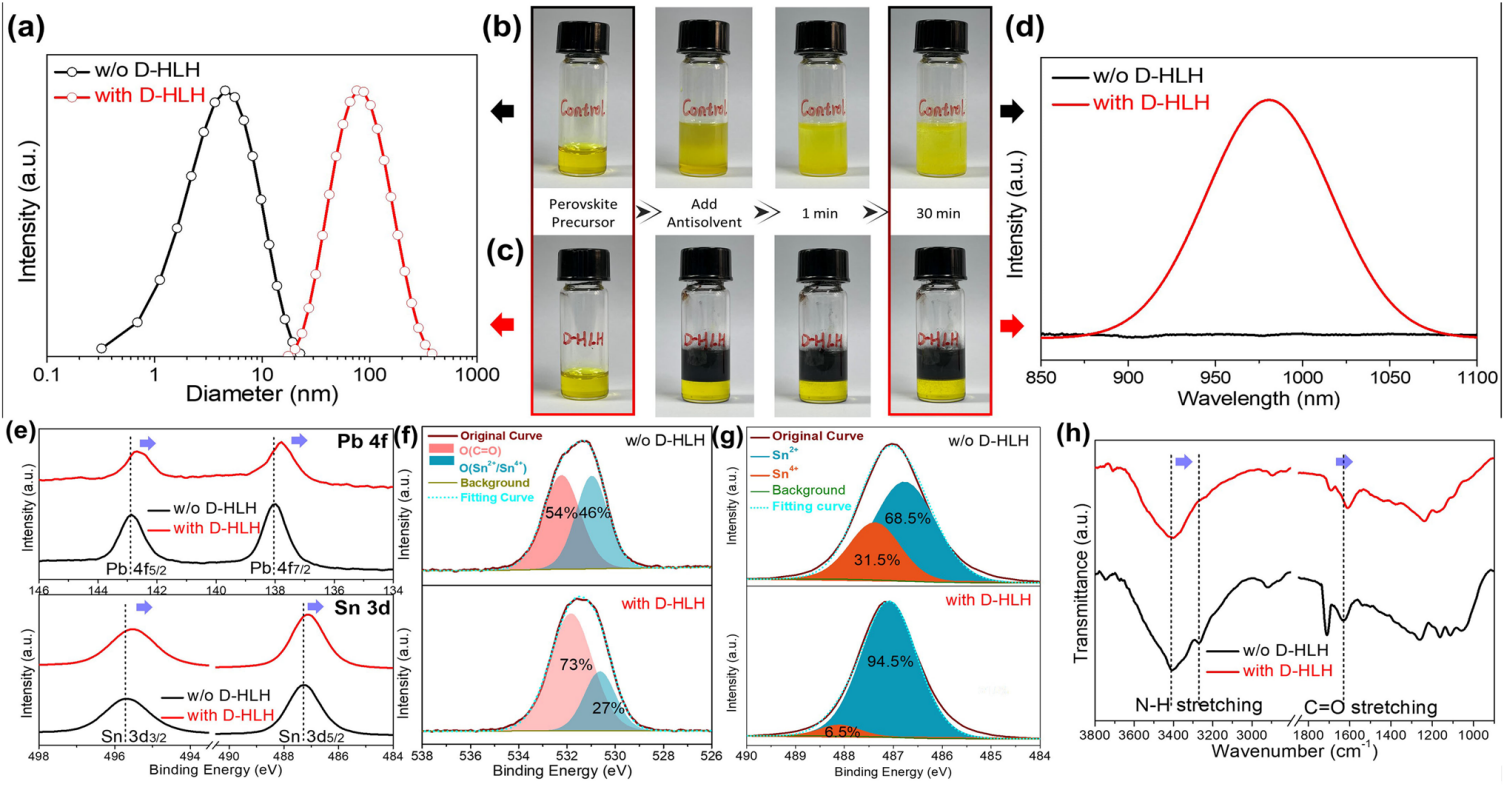
**a** DLS measurements of the colloidal sizes of the perovskite precursor solutions prepared with and without D-HLH. Photographs of perovskite precursor solutions prepared **b** without and **c** with D-HLH, taken before and after adding the antisolvent. **d** PL spectra of precursor solutions prepared with and without D-HLH. XPS spectra of perovskites prepared with and without D-HLH: **e** Pb 4*f* and Sn 3*d*, **f** O 1*s*, and **g** Sn 3*d*_5/2_. **h** FTIR spectra of perovskites prepared with and without D-HLH

Because the antisolvent extracted the intermediate phase from the solvent of the precursor solution, we initially studied the D-HLH-induced regulation of the intermediate phase based on the precursor solution. First, we used DLS to investigate the nucleation of the perovskite precursor solution (Fig. 2a). The center of the DLS peak shifted from 4.5 to 76.1 nm after the addition of D-HLH. The presence of larger colloids implied that pre-nucleation clusters had formed in the D-HLH-treated perovskite precursor solution [47, 48]. These clusters could decrease the energy barrier for nucleation during the precipitation process [49, 50]. We performed a simple experiment to visualize the formation of the intermediate phase in the precursor solution. As displayed in Fig. 2b–c, we added 0.5 mL of the antisolvent to the light-yellow precursor solutions (0.5 mL). For D-HLH-treated perovskite precursor solution, black perovskite crystals separated out from the precursor solution immediately; for the control perovskite precursor solution, a yellow-gray intermediate phase separated out near the top, but redissolved into the DMSO/DMF solvent after standing for 30 min. PL spectra verified the presence of black perovskite crystals in the precursor solution (Fig. 2d). Thus, the introduction of D-HLH appeared to lower the supersaturated concentration of the perovskite phase in the precursor solution. Therefore, the D-HLH-induced pre-nucleation process lowered the energy barrier for nucleation of the perovskite phase and facilitated the rapid nucleation of the perovskite phase after treatment with the antisolvent.

We suspected that the polar functional groups of D-HLH played a role in accelerating the formation of the preformed perovskite nuclei. We applied X-ray photoelectron spectroscopy (XPS) to measure changes in the binding energies of the perovskite components. For Pb^2+^ and Sn^2+^ ions (Fig. 2e, Table S2), addition of D-HLH caused the Pb 4*f*_7/2_ and Pb 4*f*_5/2_ peaks and the Sn 3*d*_3/2_ and Sn 3*d*_5/2_ peaks to all move to lower binding energies, suggesting that the ester group in D-HLH became anchored to the Pb^2+^ and Sn^2+^ ions through strong Pb–O and Sn–O bonds. The variations in the O and Sn contents associated with the notorious Sn^4+^ vacancies are revealed in Fig. 2f–g. Here, we fitted the O 1*s* XPS peaks to composites featuring organic (O_org_) and inorganic (O_in_) components; Table S3 provides the detailed peaks assignments. For the control sample, the contents of O_org_ and O_in_ were 54% and 46%, respectively; the contents of Sn^2+^ and Sn^4+^ were 68.5% and 31.5%, respectively. After adding D-HLH, the contents of O_org_ and Sn^2+^ increased to 73% and 94.5%, respectively, while those of O_in_ and Sn^4+^ decreased to 27% and 6.5%, respectively. These data suggest that strong Sn–O bonds formed between D-HLH and the Sn^2+^ ions, thereby inhibiting the Sn^2+^ oxidation process. Moreover, we performed a simple verification experiment (Fig. S2): after exposing the precursor solutions to the air, the pristine solution changed color from light-yellow (Sn^2+^) to deep-red (Sn^4+^) over time, whereas the D-HLH-treated solution maintained its color even after being exposed to air for 20 min. Thus, strong interactions must have existed between D-HLH and the Sn^2+^ component, sufficient to inhibit the generation of Sn^4+^ even under continuous oxygen penetration. To verify that interactions existed between D-HLH and the other perovskite components, we recorded FTIR spectra to search for any variations in the stretching peaks (Fig. 2h, Table S4). After adding D-HLH, both N–H and C = O stretching peaks moved to lower wavenumbers, suggesting that the ester group of D-HLH formed hydrogen bonds [–O–C(= O)···H–N] with the amino group of FA^+^, consistent with ester oxygen atoms being good hydrogen bond donors [51–53]. Furthermore, according to charge distribution measurements (Fig. S3), the positive dipoles are buried inside the molecules of DMF and DMSO, whereas that of D-HLH is exposed externally. As a result, D-HLH can solvate anions more readily [48]. Moreover, the polar amino group (N^δ–^–H^δ+^) of D-HLH and the electronegative iodide ion (I^δ–^) can form a hydrogen bond (–N–H···I^–^). Therefore, these data suggest that D-HLH could bond specifically with each perovskite component [through hydrogen bonding with organic (FA^+^) and I^–^ ions; through strong Pb–O Sn–O bonds with the inorganic ions (Pb^2+^, Sn^2+^)], then weaken the incomplete interactions between DMSO and the various components, and finally accelerate the conversion of the intermediate adducts into preformed perovskite nuclei.

We conclude that D-HLH promoted the effective conversion of the mixed intermediate phase into pure preformed perovskite nuclei after performing the antisolvent wash. Therefore, the introduction of D-HLH played a significant part in regulating the nucleation process and promoting the presence of homogeneous nucleation sites for crystal growth.

### Balancing the Nucleation and Crystallization Processes

**Fig. 3:**
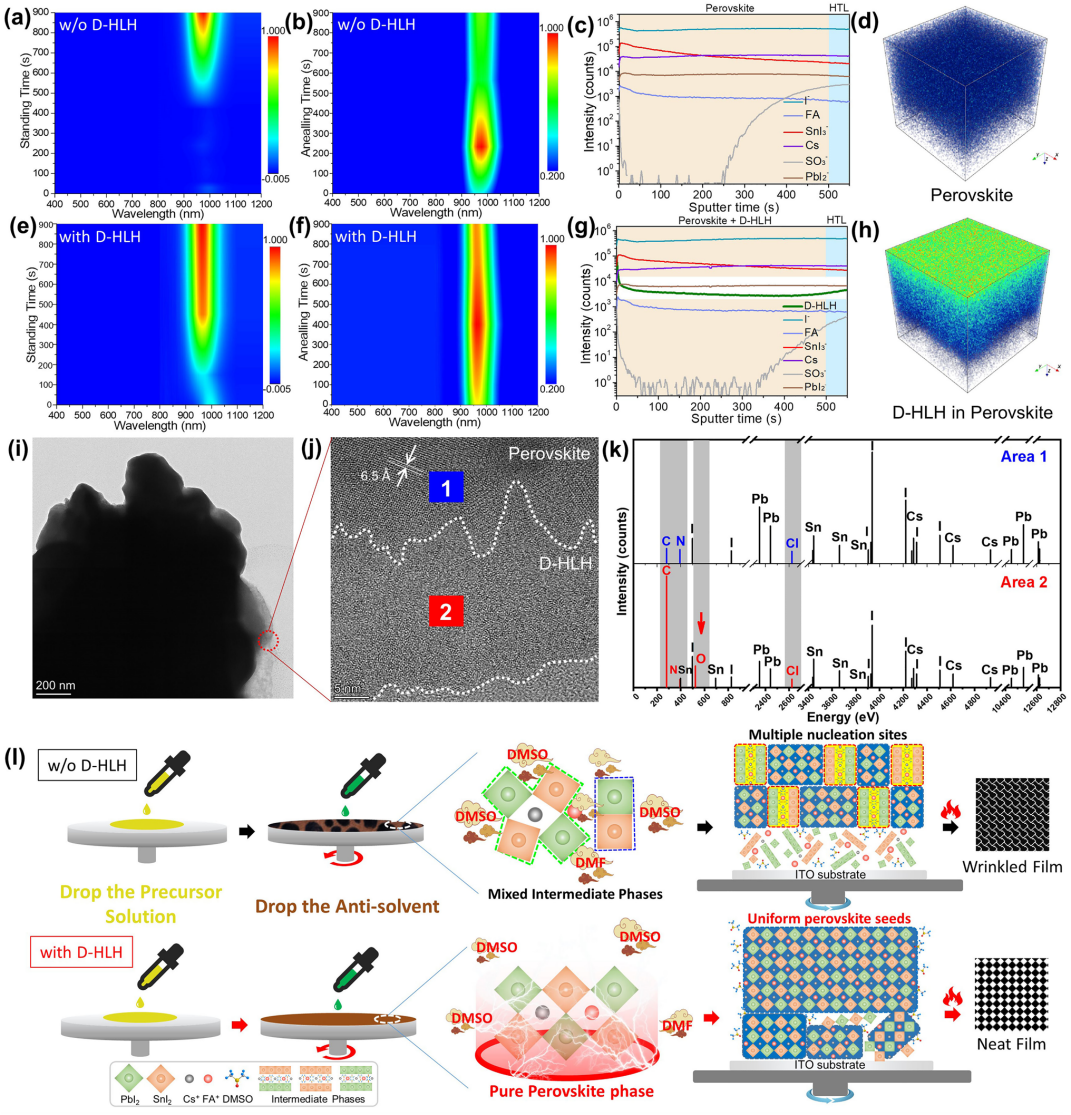
In situ PL spectral data of intermediate phase films prepared **a, b** without and **e, f** with D-HLH, after various **a, e** standing and **b, f** annealing times. **c, g** ToF–SIMS depth profiles and **d, h** 3D profiles of the perovskite films prepared **c, d** without and **g, h** with D-HLH. **i** TEM image of the perovskite prepared with D-HLH. **j** HRTEM image of the circled region in (**i**). **k** EDS spectra of the blue and red boxed regions in (**j**). **l** Schematic representation of the effects of D-HLH on the regulation of the nucleation process and on the improvement in crystalline quality

In situ PL spectroscopy is a powerful tool for studying the kinetics of nucleation and crystallization. Figure 3a, e displays the PL spectral data for the film growth process, recorded over the period from the initial spin-coating to immediately after the antisolvent wash. For sample preparation, 80 µL of the perovskite precursor was spin-coated onto the substrate. Then, 200 µL of antisolvent was dropped 5 s before the end of the spin cycle. The as-prepared film was immediately subjected to continuous PL testing. Figure 3b, f represents the film growth from the pre-annealed state to the annealed state. The as-prepared film was annealed at 100 °C and simultaneously subjected to continuous PL testing. All PL spectra were recorded from 0 to 900 s. For the control film, after treated with the antisolvent, no PL peak appeared during the initial standing time (0–500 s, Fig. 3a), with the highest PL response appearing after annealing for 10 min (Fig. 3b). We attribute this behavior to the coexistence of DMSO-containing intermediate adducts and the perovskite phase in the antisolvent-treated film. Therefore, the perovskite signal began to appear as the DMSO gradually volatilized. Furthermore, because of the presence of the DMSO-containing adducts, the highest peak for the perovskite phase appeared only after these adducts had been completely eliminated or transformed into the perovskite through annealing. In contrast, for film prepared with the addition of D-HLH, a PL peak appeared near 990 nm immediately after the antisolvent dripping process (Fig. 3e). The PL intensity grew to its highest value upon annealing (Fig. 3f). These observations confirm that the perovskite phase pre-existed in the antisolvent-treated film and that the perovskite signal emerged immediately after spin-coating. In addition, because no DMSO-containing intermediate adducts were present in the film, the highest PL intensity of the perovskite peak appeared during the early stages of the annealing process. Thus, the D-HLH-treated crystal growth process could transform the perovskite nucleus phase (of the antisolvent-treated film) directly to a highly crystalline perovskite film upon annealing, by facilitating the synchronized growth of the Cs/FA-containing Sn–Pb alloyed perovskite components having different reactivities. Therefore, thanks to the presence of D-HLH, the intermediate-phase film was directly nucleated to form pure preformed perovskite seeds, substantially balancing the nucleation and crystallization processes of the various components.

To investigate the distribution of D-HLH in the final perovskite film, we employed ToF–SIMS to determine the element depth profile of the D-HLH-treated perovskite film (Fig. 3c, d, g and h). We found that D-HLH was distributed uniformly in the whole perovskite film, as could be visually identified in corresponding 3D profiles (Fig. 3h). For further verification, we used high-resolution transmission electron microscopy (HRTEM) to discern the different lattices of the organic and inorganic components (Fig. 3i, j). An interplanar spacing of 6.5 Å in regions having a regular lattice arrangement matched well with that of the (200) plane of cubic 3D perovskite [54, 55]. Thus, the disordered lattice distribution area marked by dashed lines corresponded to organic molecules. To further identify the component presents in these regions, we employed energy dispersive X-ray spectrometry (EDS) to test the areas 1 (blue box) and 2 (red box) marked in Fig. 3j. Figure 3k reveals that the carbon (C) content in area 2 was much higher than that in area 1, and that oxygen (O) appeared in area 2 whereas no such signal appeared in area 1. Thus, the signals for C and O atoms in area 2 arose from the presence of D-HLH, confirming that the disordered lattice region in the perovskite film contained D-HLH. Therefore, all of our measurements verified that D-HLH was distributed homogeneously throughout the perovskite films, ensuring sufficient regulation of the crystal growth process. Furthermore, the D-HLH component was embedded mainly in the grain boundaries (Fig. 3j); therefore, the D-HLH-induced specific bonding might have passivated the defects present in the final films. We discuss this matter further below.

In summary, there were two distinct crystal growth processes, as displayed in Fig. 3l. Because of incomplete complexation between DMSO and the various perovskite components, including the highly reactive ions Cs^+^ and Sn^2+^, an asynchronous nucleation process occurred. As a result, the coexistence of intermediate adducts and a perovskite phase, in the form of a mixed intermediate phase, occurred after quenching with the antisolvent. This mixed intermediate phase led directly to the aggregated crystallization process, forming a poorly crystallized film. The introduction of D-HLH resulted in specific bonding with each perovskite component, through either hydrogen bonding [–O–C(= O)**···**H–N, –N–H**···**I^–^] or strong Pb–O and Sn–O bonds. These interactions substantially weakened the incomplete complexation effects induced by DMSO and facilitated fast nucleation of the perovskite phase, thereby balancing the nucleation process of each component, resulting in purely preformed perovskite nuclei as homogeneous nucleation sites. Ultimately, we obtained perovskite films having much larger grains and improved crystalline quality.

### Photovoltaic Performance of Single and Tandem Devices Prepared with D-HLH Treatment

**Fig. 4:**
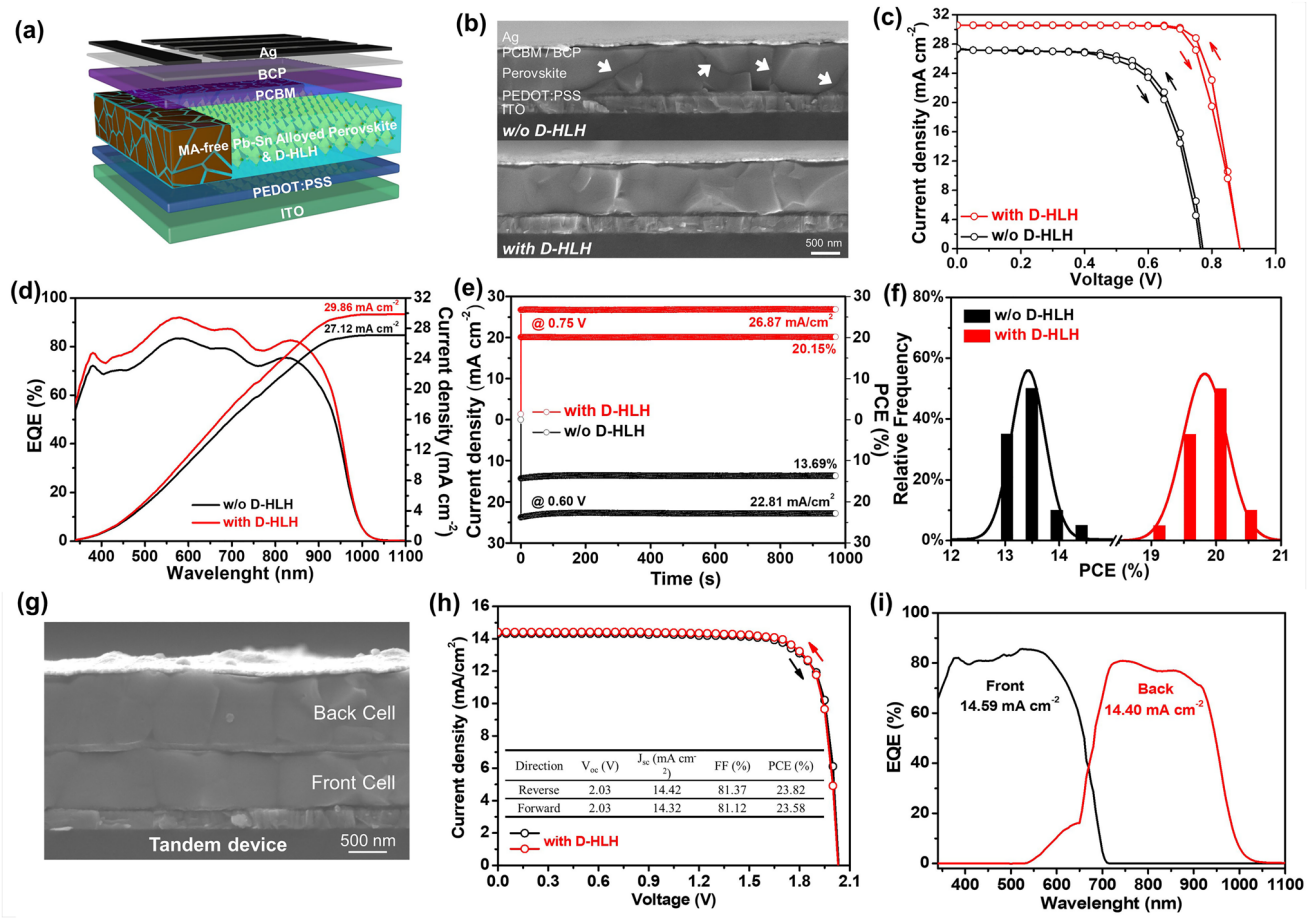
**a** Device structure of MA-free Sn–Pb alloyed PSCs prepared with D-HLH treatment. **b** Cross-sectional SEM images of devices prepared with and without D-HLH treatment. **c** J–V curves of champion PSCs, recorded in both forward and reverse scans under AM 1.5 G illumination (100 mW cm^–2^). **d** EQE spectra of the corresponding devices. **e** Steady-state current densities and corresponding PCEs of champion devices (at 0.70 V for the PSC prepared with D-HLH treatment; at 0.60 V for the pristine PSC). **f** Performance distribution diagram for 20 devices in one batch. **g** Cross-sectional SEM image of tandem device prepared with D-HLH treatment. h J–*V* curves of champion tandem devices, recorded in both forward and reverse scans under AM 1.5 G illumination (100 mW cm^–2^). **i** EQE spectra of the tandem device prepared with D-HLH treatment

Having investigated the morphology and crystalline quality of the control and D-HLH-treated films, we studied the effect of D-HLH on the photovoltaic performance of corresponding PSCs. We fabricated inverted devices having the architecture indium tin oxide (ITO)/poly(3,4-ethylenedioxythiophene):polystyrenesulfonate (PEDOT:PSS)/Cs_0.25_FA_0.75_Pb_0.6_Sn_0.4_I_3_ (prepared with or without D-HLH)/[6, 6]-phenyl-C_61_-butyric acid methyl ester (PCBM)/bathocuproine (BCP)/Ag. Figure 4a displays a schematic representation of the device structure. Figure 4b presents cross-sectional SEM images of these devices. Fractures and delamination (indicated by white arrows) appeared in the perovskite layer of the control device. In contrast, the D-HLH-treated film had a compact and dense morphology, with grains expanded uniformly to several micrometers along the substrate. We suspected that this continuous and complete perovskite layer would facilitate the generation and transmission of charge carriers.

Figure S4 displays the typical current density–voltage (*J*–*V*) curves of PSCs prepared using different concentrations of D-HLH; Table S5 lists the corresponding photovoltaic parameters. Compared with the champion PCE of 14.51% for the control device, the PCE of the device prepared with 5 mg mL^–1^ D-HLH had increased to 18.76%. A higher concentration of D-HLH was, however, harmful to the device performance. When its concentration reached 20 mg mL^–1^, the PCE of the device fell to 13.34%. Figure 4c presents the best *J*–*V* curves of the PSCs prepared with or without D-HLH treatment, recorded from both reverse and forward scans. The PSC modified with 10 mg mL^–1^ D-HLH provided a reverse-scan PCE of 21.61%, with a short-circuit current density of 30.56 mA cm^–2^, an open-circuit voltage of 0.88 V, and an FF of 80.36%. Its forward-scan PCE was 21.04%, with negligible hysteresis. This could attribute to the uniform crystal crystallization of perovskite films with improved qualities promoted by the D-HLH treatments. In comparison, the control device provided reverse- and forward-scan PCEs of 14.51% and 14.07%, respectively. Table S6 lists the detailed parameters. We attribute the significant improvement in performance of the D-HLH-treated device mainly to its higher values of *V*_oc_ (0.88 V) and FF (80.36%) relative to those of the control device (0.78 V and 70.91%, respectively). Figure 4d presents the external quantum efficiency (EQE) and integrated current density of the D-HLH-treated device. The integrated value of *J*_sc_ (29.86 mA cm^–2^) was consistent with the value of *J*_sc_ obtained from the *J*–*V* curves (30.56 mA cm^–2^). Figure 4e displays the steady-state output current densities and efficiencies of these devices, measured at their fixed MPP voltages. After continuous exposure to 1-sun illumination for over 950 s, the device prepared with D-HLH maintained a steady-state value of *J*_sc_ of 26.87 mA cm^–2^, corresponding to a stabilized efficiency of 20.15%, much higher than that of the control device (13.69%). Figure 4f provides efficiency histograms constructed from 20 control and D-HLH-treated devices; Fig. S5 displays the corresponding photovoltaic parameters, indicating that the D-HLH-treated devices possessed enhanced device reproducibility. We conclude that devices modified with D-HLH had a conspicuous improvement in photovoltaic performance and reproducibility.

Motivated by the improved quality of the MA-free Pb–Sn alloyed perovskite films prepared with D-HLH, we fabricated monolithic all-perovskite tandem solar cells with the aim of further improving the photovoltaic performance. The wide-band-gap front subcell had a composition of Cs_0.05_FA_0.8_MA_0.15_Pb(I_0.6_Br_0.4_)_3_ [56, 57]. The tandem device had the device structure ITO/NiO_x_/Cs_0.05_FA_0.8_MA_0.15_Pb(I_0.6_Br_0.4_)_3_/PCBM/SnO_2_/Au/PEDOT:PSS/Cs_0.25_FA_0.75_Pb_0.6_Sn_0.4_I_3_/PCBM/BCP/Ag (Fig. 4g). The SnO_2_ layer was deposited through atomic layer deposition at low temperature, using a reported procedure [58]. The thickness of front and back subcells were approximately 490 and 1000 nm, respectively. Figure 4h presents the *J*–*V* curves of the D-HLH tandem devices, recorded from both forward and reverse scans. Table S7 summarizes the corresponding photovoltaic parameters. The best-performing D-HLH-treated tandem device exhibited an impressively high PCE of 23.82% in the reverse scan, with a high value of *V*_oc_ of 2.03 V, a value of *J*_sc_ of 14.42 mA cm^–2^, and a high FF of 81.37%. Its forward-scan PCE was 23.58%, with negligible hysteresis. The integrated values of *J*_sc_ of the wide- and narrow-band-gap subcells, determined from their EQE spectra (Fig. 4i), were 14.59 and 14.40 mA cm^–2^, respectively, in good agreement with the values of *J*_sc_ determined from the *J*–*V* measurements. From 20 fabricated D-HLH-treated tandem devices, we obtained an average PCE of 23.38% (Fig. S6). The narrow PCE distribution highlights the excellent reproducibility of the D-HLH-treated tandem cells. We conclude that a remarkable improvement in the photovoltaic properties of tandem devices can also be realized through D-HLH treatment, because of their MA-free narrow-band-gap Sn–Pb subcells.

**Fig. 5:**
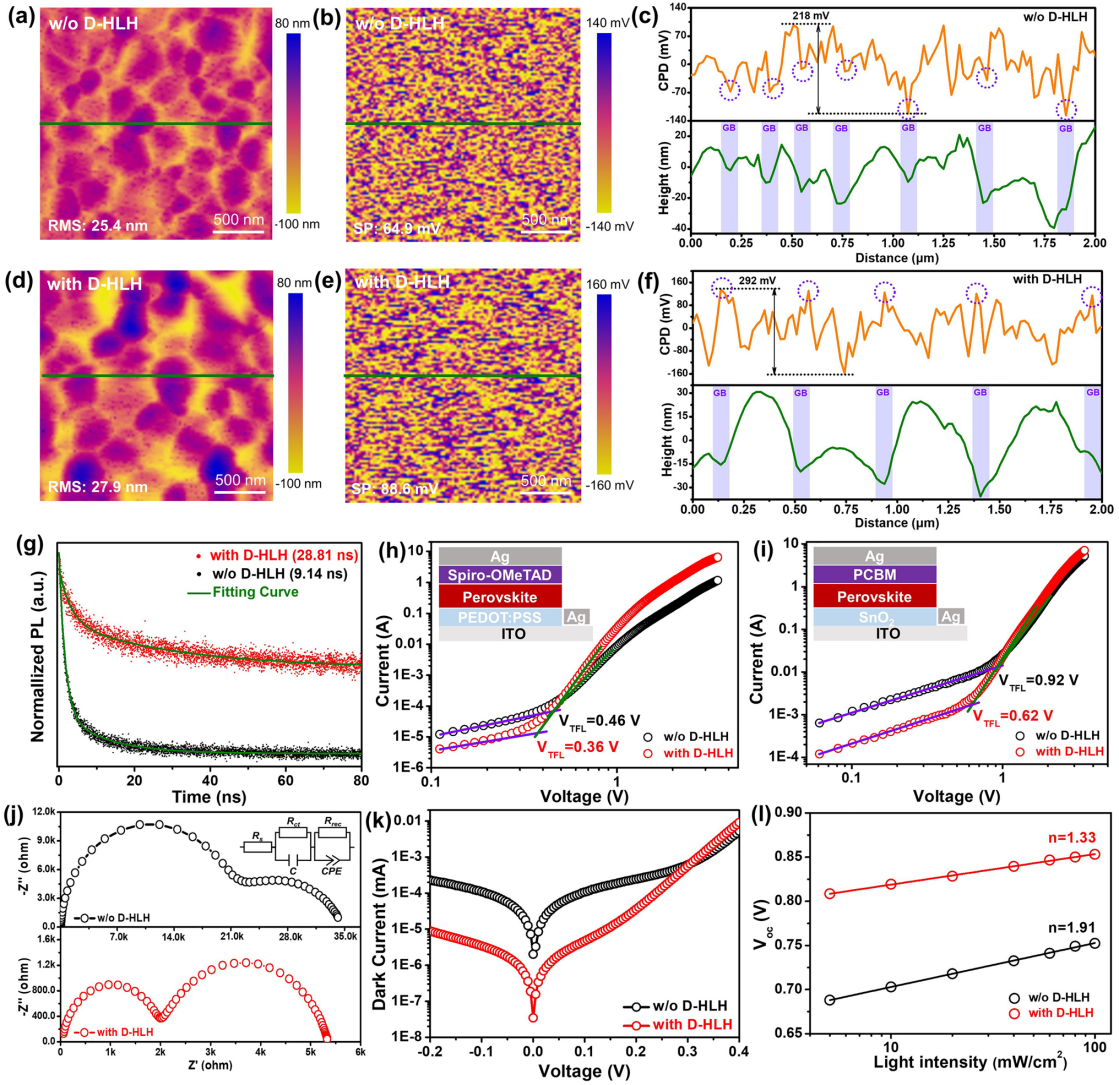
**a, d** Morphology and **b, e** CPD maps (2 × 2 μm^2^) of perovskite films prepared **a, b** without and **d, e** with D-HLH. Relative *V*_CPD_ line scan spectra of perovskite films prepared **c **without and **f** with D-HLH, extracted from the corresponding maps. **g** Normalized TRPL decay transient spectra of perovskite films grown on glass substrates. Structures and *J*–*V* plots of **h** hole- and **i** electron-only devices, for direct measurement of trap densities. **j** Nyquist plots of the PSCs. **k** Dark current–*V* curves of PSC devices. **l** Plots of *V*_oc_ with respect to light intensity, and fitting curves, of the PSCs

To identify the inherent mechanism behind the enhanced photovoltaic performance of the D-HLH-treated PSCs, we used atomic force microscopy (AFM) and Kelvin probe force microscopy (KPFM) to study the morphologies and electrical properties between the grain surfaces (GSs) and grain boundaries (GBs). Figure 5a–f displays the results. The control film provided a relatively uniform contact potential difference (*V*_CPD_) image. We estimated the value of *V*_CPD_ between the GSs and GBs to be 218 mV. Because the GBs had a relatively low potential (indicated by the purple circle in Fig. 5c), the charge carriers tended to be trapped by the GBs, thereby increasing the leakage current in the device [59]. With the addition of D-HLH, the value of *V*_CPD_ along the GBs increased significantly, relative to that of the adjacent GSs, reaching 292 mV (Fig. 5f). This finding demonstrates that films prepared without wrinkles can have significantly fewer surface defects [60]. Moreover, we also attribute this enhancement to the uniform distribution of D-HLH in the perovskite film, as verified using TOF–SIMS and HRTEM (Fig. 3h, j), thus specific bonding induced by D-HLH could passivate the defects existing in GBs. Therefore, by optimizing the crystallization process of the MA-free Sn–Pb alloyed perovskite, the electrical properties of the GSs and GBs were greatly improved, resulting in efficient charge transport.

Next, we studied the passivation effect of the D-HLH-treated perovskite films. We recorded time-resolved photoluminescence (TRPL) spectra to investigate the carrier lifetimes inside these films (Fig. 5g). The normalized TRPL spectra were fitted well using a biexponential fitting model; Table S8 lists the detailed parameters. The average recombination lifetime increased from 9.14 to 28.81 ns (almost threefold) after incorporating D-HLH, suggesting that the number of defect states or trap densities within the films had decreased substantially, representing the improved charge extraction performance at the D-HLH modified perovskite interfaces, contributing to the enhancement of the device’s *V*_oc_ and FF. Furthermore, we measured the space charge limited current (SCLC) to quantify the electron and hole trap densities in the control and D-HLH-modified perovskite films (Fig. 5h, i). The hole-only device had the structure ITO/PEDOT:PSS/perovskite/spiro-OMeTAD/Ag; the electron-only device had the structure ITO/SnO_2_/perovskite/PCBM/Ag. The hole trap density decreased from 6.51 × 10^15^ cm^–3^ for the control sample to 5.09 × 10^15^ cm^–3^ for D-HLH-modified film, while the electron trap concentration decreased accordingly from 1.29 × 10^16^ to 8.76 × 10^15^ cm^–3^. The lower hole and electron trap densities in the D-HLH-modified perovskite film indicate that this treatment considerably minimized the presence of defects within the film, consistent with the TRPL data, contributing to the enhanced value of *V*_oc_. Moreover, we used electrochemical impedance spectroscopy (EIS) to identify the charge transfer properties at the device interface and within the perovskite (Fig. 5j). Table S9 lists the fitting parameters of the equivalent circuit. As expected, the charge transfer resistance was lower for the D-HLH-modified PSC, while its carrier recombination resistance was much higher. Thus, treatment with D-HLH enhanced the degree of carrier extraction from the photoactive perovskite layer to the transporting layers, presumably because of the suppression of defects or traps. Moreover, we measured the saturation dark currents for these integrated PSCs to evaluate the charge recombination according to the diode law (Fig. 5k). The dark currents of the control and D-HLH-modified devices were 2.01 × 10^–6^ and 3.44 × 10^–8^ mA, respectively. The almost two-orders-of-magnitude decrease in the dark current of the D-HLH-treated device could further lead to effective suppression of charge recombination and Sn^4+^ in the perovskite film, contributing to its higher value of *V*_oc_. To examine the charge extraction and recombination processes in the control and D-HLH-modified devices, we measured the *J*–*V* curves of the devices under light intensities ranging from 5 to 100 mW cm^–2^. Figure 5l reveals that the value of *V*_oc_ increased monotonically with respect to the logarithm of the light intensity. The D-HLH-treated device realized an ideal value (*n*) of 1.33, whereas it was 1.91 for the control. The lower value of *n* for the D-HLH-treated device indicates a decrease in trap-assisted recombination, consistent with the enhanced carrier lifetime observed using TRPL [61, 62]. Consequently, all of these observations reveal that treatment with D-HLH could substantially decrease the number of trap densities and enhance the charge extraction in the MA-free Sn–Pb alloyed perovskite layer, resulting in enhanced photovoltaic performance.

### Stability Testing of Single and Tandem Devices Prepared with D-HLH Treatment

**Fig. 6:**
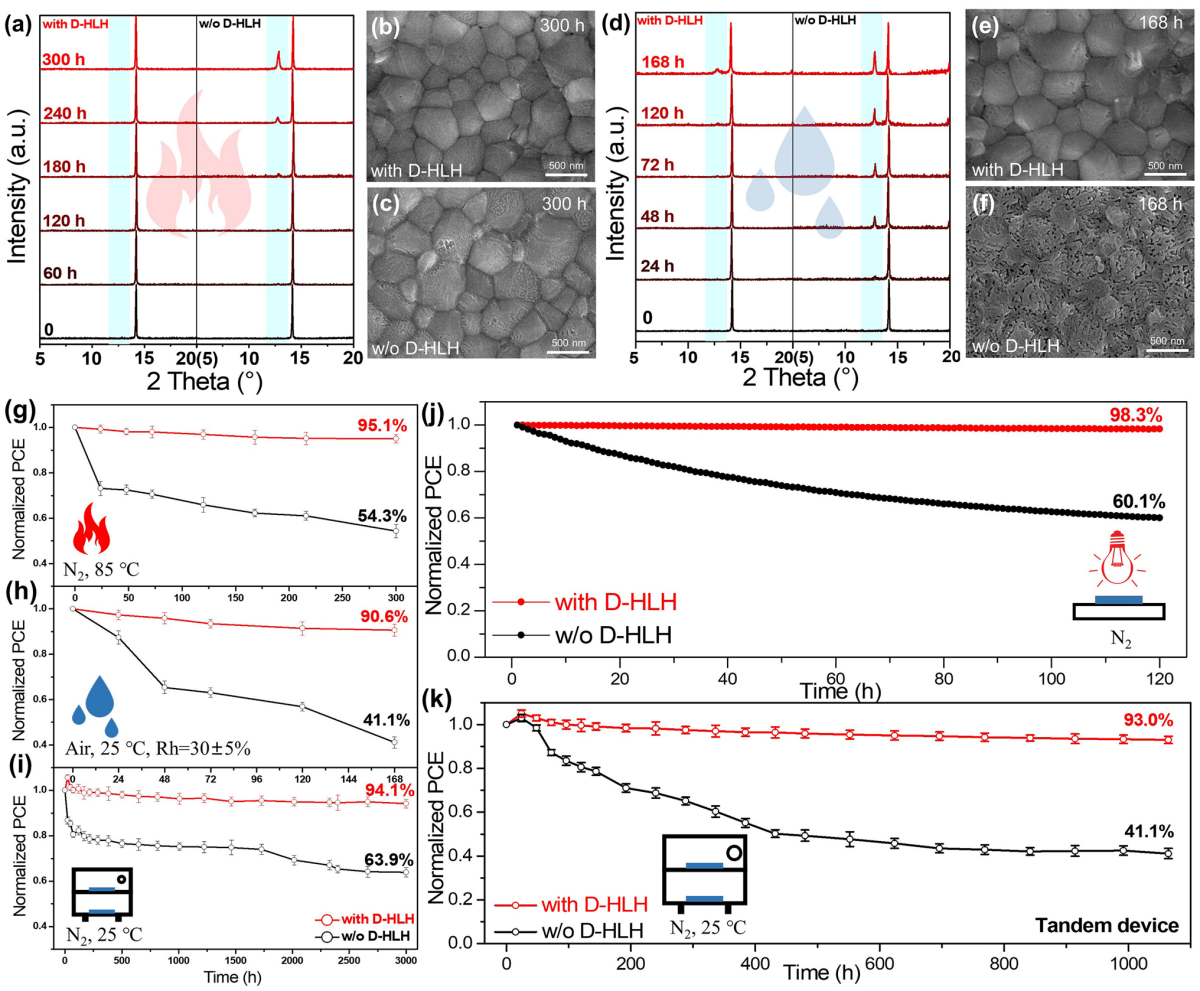
**a–c, g** Thermal stability measurements: **a** XRD spectra of perovskite films prepared with or without D-HLH; **b, c** SEM images of perovskite films prepared **b** with and **c** without D-HLH; and **g** normalized PCEs of PSC prepared with and without D-HLH. **d–f, h** Humidity stability measurements: **d** XRD spectra of perovskite films prepared with and without D-HLH; **e, f** SEM images of perovskite films prepared **e** with and **f** without D-HLH; and **h** normalized PCEs of PSCs prepared with and without D-HLH. **i, k** Shelf stability of **i** single and **k** tandem devices prepared with and without D-HLH. **j** MPP tracking of unencapsulated single devices prepared with and without D-HLH treatment, measured in a N_2_ glove box under 1-sun AM 1.5G illumination. The initial PCEs of the single devices prepared with and without D-HLH treatment were 21.15% and 13.68%, respectively

In addition to efficiency, stability is another important parameter when evaluating device performance. Therefore, to investigate the potential of using MA-free Sn–Pb alloyed PSCs in real applications, we tested their thermal, humidity, shelf-storage, and operational stabilities. The samples that were susceptible to light degradation were stored in the dark. To measure the thermal stability, we aged unencapsulated perovskite films and single-junction devices on a hotplate at 85 °C in N_2_-filled glove box (H_2_O, 0.1 ppm; O_2_, 0.1 ppm) for 300 h (Fig. 6a–c, g). XRD patterns revealed that the signal for the PbI_2_/SnI_2_ phase in the control film appeared initially at a diffraction angle of 12.8° and gradually increased during the aging process, whereas the D-HLH-treated perovskite film maintained its high crystallinity [63]. After aging for 300 h, many cracks were distributed on the crystals on the surface of the control film (Fig. 6c), whereas no obvious changes had occurred to the D-HLH-treated film (Fig. 6b). Furthermore, our device that had been subjected to D-HLH treatment retained 95.1% of its initial efficiency after 300 h, whereas the control device retained only 54.3% of its initial performance. The thermal stability of the D-HLH-treated Cs_0.25_FA_0.75_Pb_0.6_Sn_0.4_I_3_ PSCs was considerably improved when compared with that of the control PSCs, presumably because of the enhanced crystal growth and the highly crystalline film.

Next, we measured the humidity stability of single-junction devices, without encapsulation, in ambient air (*T* = 25 °C; RH = 30 ± 5%) for 168 h (Fig. 6d–f, h). The XRD pattern of the control film features a high-intensity signal for the PbI_2_/SnI_2_ phase after only 48 h of exposure to the ambient air; that of the D-HLH-modified film underwent no change, even for 168 h. Furthermore, the D-HLH-treated film surface remained clean after exposure to the ambient air for 168 h, whereas numerous holes, corroded by water and oxygen, were distributed on the control film. Moreover, our device treated with D-HLH retained 90.6% of its initial PCE after 168 h, much higher than that of the control device (41.1%). We attribute this improvement to the enhanced crystallinity and lower number of trap densities, thereby hindering the adsorption of water and oxygen [64].

We tested the shelf-storage stability for both single-junction and tandem PSCs, without encapsulation, in N_2_-filled glove box (maintained at 25 °C) (Fig. 6i, k). Under these storage conditions, environmental factors were absent, so the main degradation would have resulted from internal factors, such as any unstable perovskite components containing Sn^4+^ and the acid nature of PEDOT:PSS [65–68]. Here, our single-junction device modified with D-HLH retained 94.1% of its initial PCE after 3000 h, while the tandem device maintained 93% of its PCE after more than 1000 h; in contrast, the control single-junction and tandem devices retained only 63.9% and 41.1%, respectively, of their initial PCEs. Because the D-HLH molecules were distributed uniformly in the perovskite films, as observed using TOF–SIMS, they could form strong Sn–O bonds that inhibited the slow oxidation of the Sn^2+^ species.

Finally, we found that the D-HLH-treated Cs/FA-based Sn–Pb alloyed PSC exhibited much improved operational stability. Figure 6j reveals that the D-HLH-treated unencapsulated single-junction device retained 98.3% of its original efficiency after MPP tracking for 120 h under continuous 1-sun illumination with an LED light source (maintained at 25 °C). The control device retained only 60.1% of its initial PCE after 120 h. This significantly improved stability originated from the enhanced crystal growth induced by the presence of D-HLH. Overall, the crystal quality of the MA-free Sn–Pb alloyed perovskite determined the stabilities of the devices.

## Conclusion

We have realized wrinkleless MA-free Sn–Pb alloyed perovskite films by reconstructing its nucleation process without forming a mixed intermediate phase. First, we found that the wrinkles on the pristine MA-free Sn–Pb alloyed perovskite films resulted mainly from the presence of mixed intermediate and perovskite phases after performing the antisolvent wash. We then introduced D-HLH to achieve specific bonding with the perovskite components, weakening the incomplete complexation reaction between the polar aprotic solvents (e.g., DMSO) and the organic (FAI) and inorganic (CsI, PbI_2_, SnI_2_) components. This additive promoted the complete transformation of the mixed intermediate phase into pure preformed perovskite nuclei, thereby inducing a homogeneous nucleation process for effective regulation of the crystallization kinetics. As a result, the optimized MA-free Sn–Pb single-junction device provided a new record PCE of 21.61%, along with increases in the values of *V*_oc_ (from 0.78 to 0.88 V) and the FF (from 70.91% to 80.36%). Furthermore, a corresponding tandem device realized a high PCE of 23.82%. The thermal, humidity, shelf-storage, and operational stabilities of the devices were all improved significantly, owing to the high crystallinity of the modified perovskite film and effective inhibition of the oxidation of Sn^2+^ species. In short, by enhancing the homogeneous nucleation of the intermediate phase, we have achieved significant improvements in film quality and substantial enhancements in both the efficiency and stability of corresponding devices. These findings for MA-free Sn–Pb alloyed perovskites suggest that alloyed perovskite systems are one step closer to practical application and commercialization.

## Supplementary Information

Below is the link to the electronic supplementary material.

Supplementary file1 (PDF 594 KB)

## References

[1] Min H, Lee DY, Kim J, Kim G, Lee KS (2021). Perovskite solar cells with atomically coherent interlayers on SnO_2_ electrodes. Nature.

[2] Hu S, Otsuka K, Murdey R, Nakamura T, Truong MA (2022). Optimized carrier extraction at interfaces for 23.6% efficient tin–lead perovskite solar cells. Energy Environ. Sci..

[3] Takahashi Y, Obara R, Lin ZZ, Takahashi Y, Naito T (2021). Charge-transport in tin-iodide perovskite CH_3_NH_3_SnI_3_: origin of high conductivity. Dalt. Trans..

[4] Noel NK, Stranks SD, Abate A, Wehrenfennig C, Guarnera S (2014). Lead-free organic-inorganic tin halide perovskites for photovoltaic applications. Energy Environ. Sci..

[5] Gu F, Ye S, Zhao Z, Rao H, Liu Z (2018). Improving performance of lead-free formamidinium tin triiodide perovskite solar cells by tin source purification. Sol. RRL.

[6] Tai Q, Guo X, Tang G, You P, Ng TW (2019). Antioxidant grain passivation for air-stable tin-based perovskite solar cells. Angew. Chem. Int. Ed..

[7] Lee SJ, Shin SS, Kim YC, Kim D, Ahn TK (2016). Fabrication of efficient formamidinium tin iodide perovskite solar cells through SnF_2_-pyrazine complex. J. Am. Chem. Soc..

[8] Liao W, Zhao D, Yu Y, Grice CR, Wang C (2016). Lead-free inverted planar formamidinium tin triiodide perovskite solar cells achieving power conversion efficiencies up to 6.22%. Adv. Mater..

[9] Ma L, Hao F, Stoumpos CC, Phelan BT, Wasielewski MR (2016). Carrier diffusion lengths of over 500 nm in lead-free perovskite CH_3_NH_3_SnI_3_ films. J. Am. Chem. Soc..

[10] Song Z, Wang C, Phillips AB, Grice CR, Zhao D (2018). Probing the origins of photodegradation in organic-inorganic metal halide perovskites with time-resolved mass spectrometry. Sustain. Energy Fuels.

[11] Juarez-Perez EJ, Ono LK, Qi Y (2019). Thermal degradation of formamidinium based lead halide perovskites into: sym-triazine and hydrogen cyanide observed by coupled thermogravimetry-mass spectrometry analysis. J. Mater. Chem. A.

[12] Juarez-Perez EJ, Ono LK, Maeda M, Jiang Y, Hawash Z (2018). Photodecomposition and thermal decomposition in methylammonium halide lead perovskites and inferred design principles to increase photovoltaic device stability. J. Mater. Chem. A.

[13] Turren-Cruz SH, Hagfeldt A, Saliba M (2018). Methylammonium-free, high-performance, and stable perovskite solar cells on a planar architecture. Science.

[14] Lang F, Shargaieva O, Brus VV, Neitzert HC, Rappich J (2018). Influence of radiation on the properties and the stability of hybrid perovskites. Adv. Mater..

[15] Liao W, Zhao D, Yu Y, Shrestha N, Ghimire K (2016). Fabrication of efficient low-bandgap perovskite solar cells by combining formamidinium tin iodide with methylammonium lead iodide. J. Am. Chem. Soc..

[16] Wang C, Song Z, Li C, Zhao D, Yan Y (2019). Low-bandgap mixed tin-lead perovskites and their applications in all-perovskite tandem solar cells. Adv. Funct. Mater..

[17] Xu G, Bi P, Wang S, Xue R, Zhang J (2018). Integrating ultrathin bulk-heterojunction organic semiconductor intermediary for high-performance low-bandgap perovskite solar cells with low energy loss. Adv. Funct. Mater..

[18] Prasanna R, Leijtens T, Dunfield SP, Raiford JA, Wolf EJ (2019). Design of low bandgap tin–lead halide perovskite solar cells to achieve thermal, atmospheric and operational stability. Nat. Energy.

[19] Lee JW, Kim DH, Kim HS, Seo SW, Cho SM (2015). Formamidinium and cesium hybridization for photo- and moisture-stable perovskite solar cell. Adv. Energy Mater..

[20] Li Z, Yang M, Park JS, Wei SH, Berry JJ (2016). Stabilizing perovskite structures by tuning tolerance factor: formation of formamidinium and cesium lead iodide solid-state alloys. Chem. Mater..

[21] Yi C, Luo J, Meloni S, Boziki A, Ashari-Astani N (2016). Entropic stabilization of mixed A-cation ABX_3_ metal halide perovskites for high performance perovskite solar cells. Energy Environ. Sci..

[22] Zhang Z, Liang J, Zheng Y, Wu X, Wang J (2021). Balancing crystallization rate in a mixed Sn–Pb perovskite film for efficient and stable perovskite solar cells of more than 20% efficiency. J. Mater. Chem. A.

[23] Xiang W, Zhang J, Liu S, Albrecht S, Hagfeldt A (2022). Intermediate phase engineering of halide perovskites for photovoltaics. Joule.

[24] Werner J, Moot T, Gossett TA, Gould IE, Palmstrom AF (2020). Improving low-bandgap tin-lead perovskite solar cells via contact engineering and gas quench processing. ACS Energy Lett..

[25] Lee JW, Kim HS, Park NG (2016). Lewis acid-base adduct approach for high efficiency perovskite solar cells. Acc. Chem. Res..

[26] Dunlap-Shohl WA, Zhou Y, Padture NP, Mitzi DB (2019). Synthetic approaches for halide perovskite thin films. Chem. Rev..

[27] Jung M, Ji SG, Kim G, Seok SI (2019). Perovskite precursor solution chemistry: from fundamentals to photovoltaic applications. Chem. Soc. Rev..

[28] Miyamae H, Numahata Y, Nagata M (1980). The crystal structure of lead (II) iodide-dimethylsulphoxide (1/2), PbI_2_ (DMSO)_2_. Chem. Lett..

[29] Wakamiya A, Endo M, Sasamori T, Tokitoh N, Ogomi Y (2014). Reproducible fabrication of efficient perovskite-based solar cells: x-ray crystallographic studies on the formation of CH_3_NH_3_PbI_3_ layers. Chem. Lett..

[30] Yang WS, Noh JH, Jeon NJ, Kim YC, Ryu S (2015). High-performance photovoltaic perovskite layers fabricated through intramolecular exchange. Science.

[31] Park B, Kedem N, Kulbak M, Lee DY, Yang WS (2018). Understanding how excess lead iodide precursor improves halide perovskite solar cell performance. Nat. Commun..

[32] Chen Z, Zhang H, Yao F, Tao C, Fang G (2020). Room temperature formation of semiconductor grade α-FAPbI_3_ films for efficient perovskite solar cells. Cell Rep. Phys. Sci..

[33] Ren Y, Zhang N, Wang Q, Zhu J, Li C (2020). Restricting δ-phase transformation of HC(NH_2_)_2_PbI_3_ via iodine-vacancy filling for efficient perovskite solar cells. Sci. China Mater..

[34] Zhou G, Wu J, Zhao Y, Li Y, Shi J (2018). Application of cesium on the restriction of precursor crystallization for highly reproducible perovskite solar cells exceeding 20% efficiency. ACS Appl. Mater. Interfaces.

[35] Qin M, Tse K, Lau TK, Li Y, Su CJ (2019). Manipulating the mixed-perovskite crystallization pathway unveiled by in situ GIWAXS. Adv. Mater..

[36] Moot T, Marshall AR, Wheeler LM, Habisreutinger SN, Schloemer TH (2020). CsI-antisolvent adduct formation in all-inorganic metal halide perovskites. Adv. Energy Mater..

[37] Jiang X, Li H, Zhou Q, Wei Q, We M (2021). One-step synthesis of SnI_2_·(DMSO)_x_ adducts for high-performance tin perovskite solar cells. J. Am. Chem. Soc..

[38] Yin M, Xie F, Chen H, Yang X, Ye F (2016). Annealing-free perovskite films by instant crystallization for efficient solar cells. J. Mater. Chem. A.

[39] Fang X, Wu Y, Lu Y, Sun Y, Zhang S (2017). Annealing-free perovskite films based on solvent engineering for efficient solar cells. J. Mater. Chem. C.

[40] Yun Y, Vidyasagar D, Lee M, Gong OY, Jung J (2021). Intermediate phase-free process for methylammonium lead iodide thin film for high-efficiency perovskite solar cells. Adv. Sci..

[41] Fateev SA, Petrov AA, Khrustalev VN, Dorovatovskii PV, Zubavichus YV (2018). Solution processing of methylammonium lead iodide perovskite from γ-butyrolactone: crystallization mediated by solvation equilibrium. Chem. Mater..

[42] Zhang L, Cao K, Qian J, Huang Y, Wang X (2020). Crystallization control and multisite passivation of perovskites with amino acid to boost the efficiency and stability of perovskite solar cells. J. Mater. Chem. C.

[43] Xiao K, Lin YH, Zhang M, Oliver RDJ, Wang X (2022). Scalable processing for realizing 21.7%-efficient all-perovskite tandem solar modules. Science.

[44] Guo X, McCleese C, Kolodziej C, Samia ACS, Zhao Y (2016). Identification and characterization of the intermediate phase in hybrid organic-inorganic MAPbI_3_ perovskite. Dalt. Trans..

[45] Rong Y, Venkatesan S, Guo R, Wang Y, Bao J (2016). Critical kinetic control of non-stoichiometric intermediate phase transformation for efficient perovskite solar cells. Nanoscale.

[46] Wang L, Wang X, Deng LL, Leng S, Guo X (2020). The mechanism of universal green antisolvents for intermediate phase controlled high-efficiency formamidinium-based perovskite solar cells. Mater. Horizons.

[47] Meng X, Li Y, Qu Y, Chen H, Jiang N (2021). Crystallization kinetics modulation of FASnI_3_ films with pre-nucleation clusters for efficient lead-free perovskite solar cells. Angew. Chem. Int. Ed..

[48] Su Y, Yang J, Liu G, Sheng W, Zhang J (2021). Acetic acid-assisted synergistic modulation of crystallization kinetics and inhibition of Sn^2+^ oxidation in tin-based perovskite solar cells. Adv. Funct. Mater..

[49] Zhang K, Wang Z, Wang G, Wang J, Li Y (2020). A prenucleation strategy for ambient fabrication of perovskite solar cells with high device performance uniformity. Nat. Commun..

[50] Gebauer D, Kellermeier M, Gale JD, Bergström L, Cölfen H (2014). Pre-nucleation clusters as solute precursors in crystallisation. Chem. Soc. Rev..

[51] Johansson A, Kollman P, Rothenberg S, McKelvey J (1974). Hydrogen bonding ability of the amide group. J. Am. Chem. Soc..

[52] R.J. Ouellette, J.D. Rawn, Structure and bonding in organic compounds. in *Organic Chemistry,* (2018) pp.1–30 doi: 10.1016/b978-0-12-812838-1.50001-3

[53] Lommerse JPM, Price SL, Taylor R (1997). Hydrogen bonding of carbonyl, ether, and ester oxygen atoms with alkanol hydroxyl groups. J. Comput. Chem..

[54] Zhan Y, Yang F, Chen W, Chen H, Shen Y (2021). Elastic lattice and excess charge carrier manipulation in 1D–3D perovskite solar cells for exceptionally long-term operational stability. Adv. Mater..

[55] Lai H, Lu D, Xu Z, Zheng N, Xie Z (2020). Organic-salt-assisted crystal growth and orientation of quasi-2D ruddlesden–popper perovskites for solar cells with efficiency over 19%. Adv. Mater..

[56] Lin Y, Chen B, Zhao F, Zheng X, Deng Y (2017). Matching charge extraction contact for wide-bandgap perovskite solar cells. Adv. Mater..

[57] Zheng Y, Wu X, Liang J, Zhang Z, Jiang J (2022). Downward homogenized crystallization for inverted wide-bandgap mixed-halide perovskite solar cells with 21% efficiency and suppressed photo-induced halide segregation. Adv. Funct. Mater..

[58] Wang Y, Ju H, Mahmoudi T, Liu C, Zhang C (2021). Cation-size mismatch and interface stabilization for efficient NiOx-based inverted perovskite solar cells with 219% efficiency. Nano Energy.

[59] Kim H, Lee JW, Han GR, Kim SK, Oh JH (2021). Synergistic effects of cation and anion in an ionic imidazolium tetrafluoroborate additive for improving the efficiency and stability of half-mixed Pb-Sn perovskite solar cells. Adv. Funct. Mater..

[60] Yun JS, Ho-Baillie A, Huang S, Woo SH, Heo Y (2015). Benefit of grain boundaries in organic-inorganic halide planar perovskite solar cells. J. Phys. Chem. Lett..

[61] Qiu Y, Liang J, Zhang Z, Deng Z, Xu H (2021). Tuning the interfacial dipole moment of spacer cations for charge extraction in efficient and ultrastable perovskite solar cells. J. Phys. Chem. C.

[62] Zhu C, Niu X, Fu Y, Li N, Hu C (2019). Strain engineering in perovskite solar cells and its impacts on carrier dynamics. Nat. Commun..

[63] Klug MT, Milot RL, Milot RL, Patel JB, Green T (2022). Metal composition influences optoelectronic quality in mixed-metal lead–tin triiodide perovskite solar absorbers. Energy Environ. Sci..

[64] Liang J, Zhang Z, Xue Q, Zheng Y, Wu X (2022). A finely regulated quantum well structure in quasi-2D Ruddlesden-Popper perovskite solar cells with efficiency exceeding 20%. Energy Environ. Sci..

[65] Kim EH, Lee JH, Kim SH, Gu JH, Lee D (2021). A-Site effect on the oxidation process of Sn-halide perovskite: first-principles calculations. J. Phys. Chem. Lett..

[66] Cameron J, Skabara PJ (2022). The damaging effects of the acidity in PEDOT:PSS on semiconductor device performance and solutions based on non-acidic alternatives. Mater. Horizons.

[67] Xu Y, Lin Z, Wei W, Hao Y, Liu S (2022). Recent progress of electrode materials for flexible perovskite solar cells. Nano-Micro Lett..

[68] Wu T, Liu X, Luo X, Segawa H, Tong G (2022). Heterogeneous FASnI_3_ absorber with enhanced electric field for high-performance lead-free perovskite solar cells. Nano-Micro Lett..

