# Supplementary material for: Resolving Mixed Intermediate Phases in Methylammonium-Free Sn–Pb Alloyed Perovskites for High-Performance Solar Cells
Source: Nanomicro Lett. 2022 Aug 16;14:165. doi: 10.1007/s40820-022-00918-1 (PMC9381682; doi:10.1007/s40820-022-00918-1)
Supplement: Supplementary file 1 — Supplementary file1 (PDF 594 KB) [file 40820_2022_918_MOESM1_ESM.pdf]

Supporting Information for

Resolving Mixed Intermediate Phases in Methylammonium-Free Sn-Pb Alloyed Perovskites for High-Performance Solar Cells

Zhanfei Zhang[1], Jianghu Liang[1], Jianli Wang[1], Yiting Zheng[1], Xueyun Wu[1], Congcong Tian[1], Anxin Sun[1], Zhenhua Chen[2], Chun-Chao Chen[1,] *

[1]School of Materials Science and Engineering, Shanghai Jiao Tong University, Shanghai 20024, P. R. China

[2]Shanghai Synchrotron Radiation Facility (SSRF), Shanghai Advanced Research Institute, Chinese Academy of Sciences, Shanghai 201800, P. R. China

*Corresponding author. E-mail: c3chen@sjtu.edu.cn (Chun-Chao Chen)

Supplementary Figures

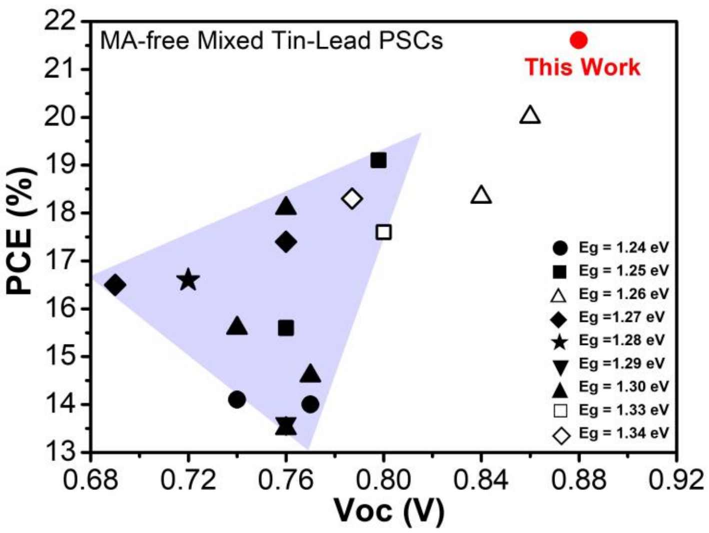

Fig. S1 Efficiencies of previously reported MA-free Pb–Sn alloyed PSCs, plotted with their the band gaps (E_g). All references for this figure are given in **Table S1**

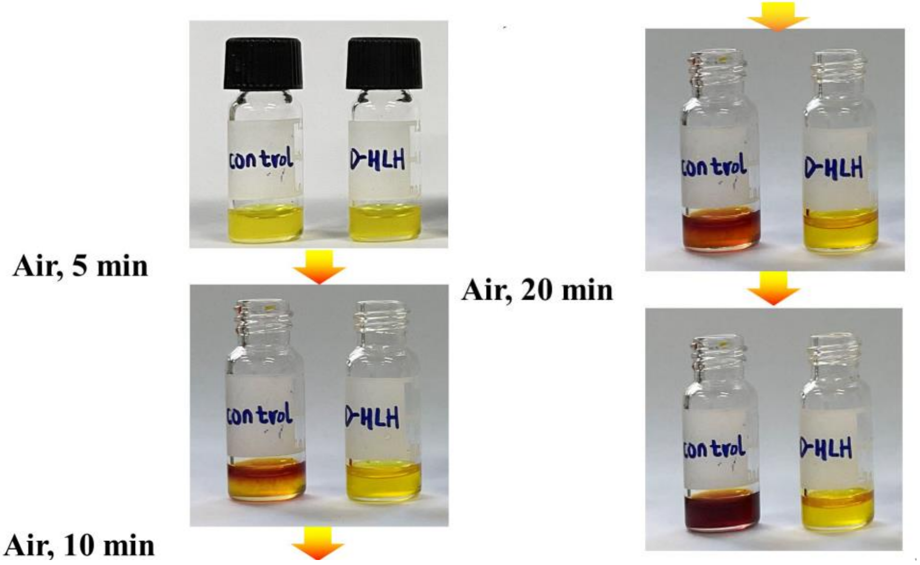

Fig. S2 Photographs of $Cs_{0.25}FA_{0.75}Pb_{0.6}Sn_{0.4}I_3$ perovskite precursor solutions, prepared with or without D-HLH, after exposure to the air for up to 20 min

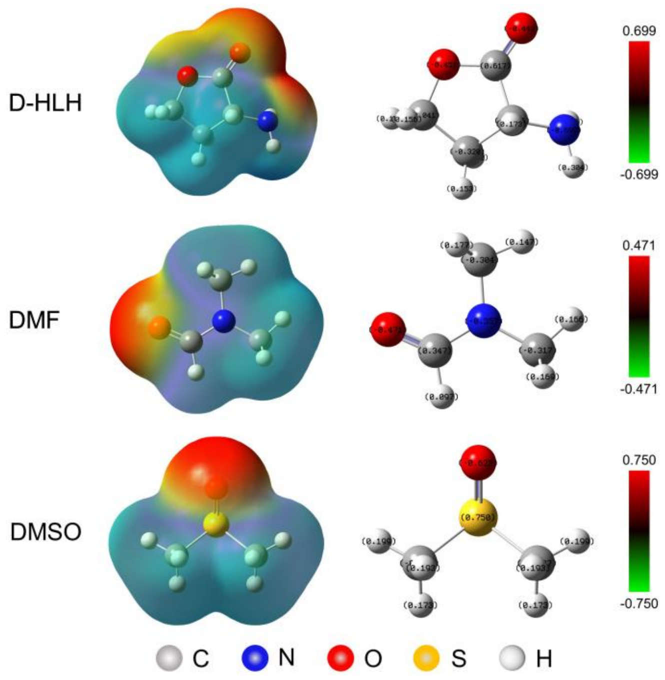

Fig. S3 (**left**) Electrostatic potentials and (**right**) charge distributions of D-HLH, DMF, and DMSO

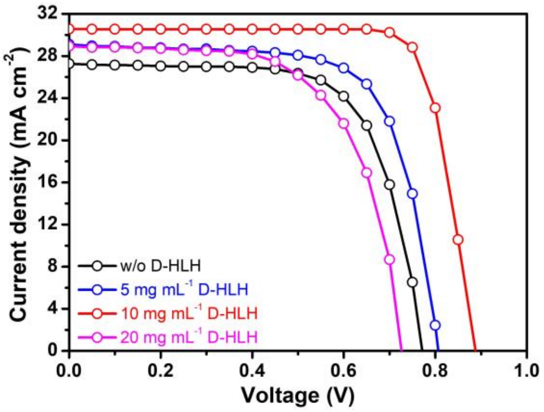

Fig. S4 *J*–*V* curves of PSCs prepared using different concentrations of D-HLH

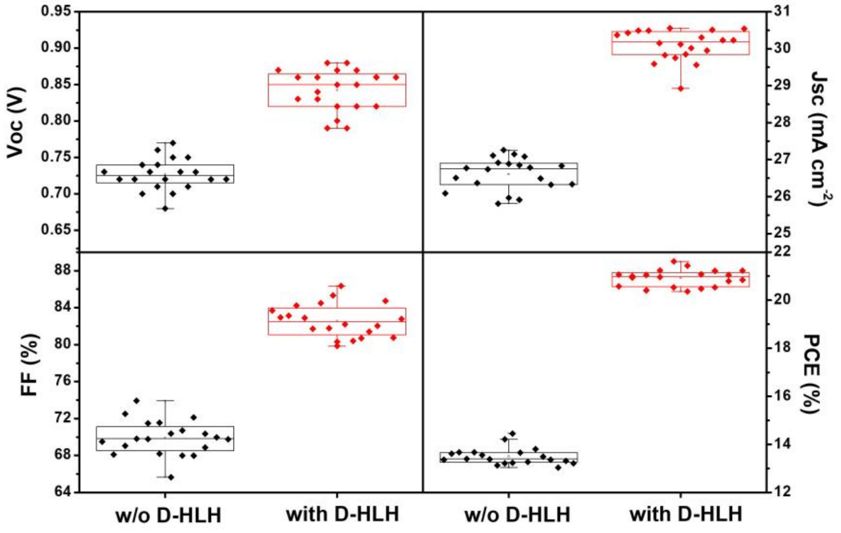

Fig. S5 Statistics of the values of V_{oc}, J_{sc}, FF, and PCE from 20 devices containing the control and D-HLH–treated perovskites

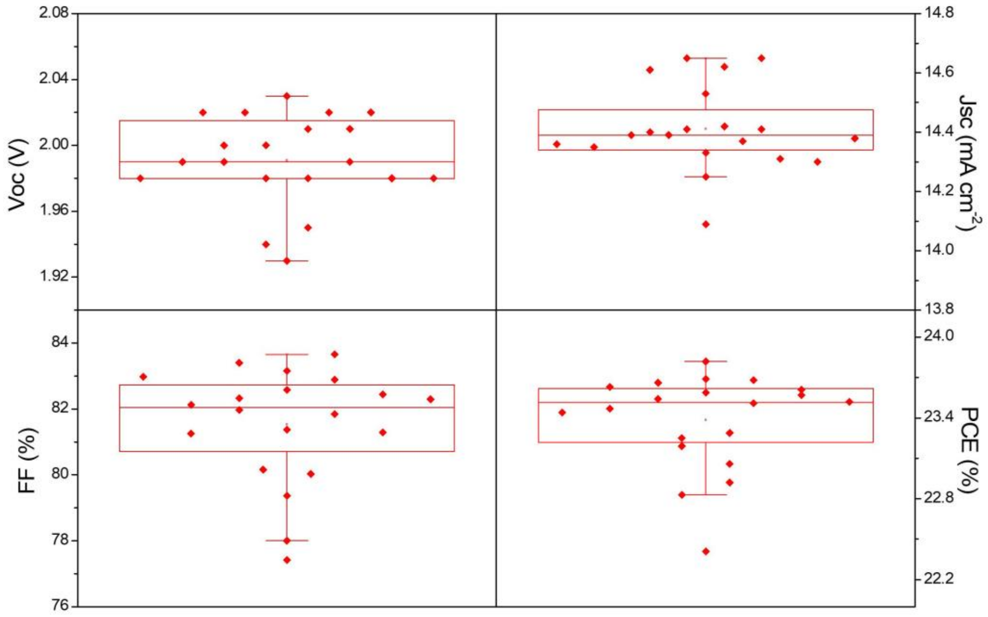

Fig. S6 Statistics of the values of V_{oc}, J_{sc}, FF, and PCE from 20 tandem devices prepared with D-HLH treatment

Table S1 Performance data of reported highly efficient MA-free Pb–Sn alloyed PSCs

**Perovskite**	***E*g (eV)**	***V*oc (V)**	***J*sc (mA cm^{-2})**	**FF (%)**	**PCE (%)**	**Stability**			**Refs.**
						Thermal (85 °C)	Light (MPP*)	Storage (N_2)	
$Cs_{0.25}FA_{0.75}Pb_{0.5}Sn_{0.5}I_3$	1.24	0.74	26.7	71	14.1	70 h, 40%; 1500 h, 30%	50 min, 90%	–	[S1]
$Cs_{0.2}FA_{0.8}Sn_{0.5}Pb_{0.5}I_3$	1.24	0.77	25.6	69	14.0				
$Cs_{0.15}FA_{0.85}Sn_{0.625}Pb_{0.375}I_3$	1.26	0.76	24.6	70	13.5				
$Cs_{0.225}FA_{0.775}Sn_{0.625}Pb_{0.375}I_3$	1.28	0.76	25.6	72	13.6	–	–	–	[S2]
$Cs_{0.2}FA_{0.8}Sn_{0.7}Pb_{0.3}I_3$	1.30	0.76	25.1	76	13.5				
$Cs_{0.3}FA_{0.7}Pb_{0.7}Sn_{0.3}I_3$	1.30	0.77	26.4	71.6	14.6	–	–	–	[S3]
$Cs_{0.25}FA_{0.75}Pb_{0.5}Sn_{0.5}I_3$	1.25	0.76	27.6	74	15.6	325 h, 82%	30 h, 100%	–	[S4]
$Cs_{0.3}FA_{0.7}Pb_{0.7}Sn_{0.3}I_3$	1.30	0.74	25.89	81.4	15.6	–	–	288 h, 98.3%	[S5]
$Cs_{0.25}FA_{0.75}Sn_{0.5}Pb_{0.5}I_3$	1.29	0.72	30.8	74.95	16.6	600 h, 50%	1000 h, 90%	–	[S6]
$Cs_{0.25}FA_{0.75}Sn_{0.5}Pb_{0.5}I_3$	1.27	0.69	31.7	76	16.5	–	–	–	[S7]
$Cs_{0.15}FA_{0.85}Sn_{0.3}Pb_{0.7}I_3$	1.33	0.80	28.7	73.5	17.6	–	–	–	[S8]
$Cs_{0.15}FA_{0.85}Sn_{0.5}Pb_{0.5}I_3$	1.30	0.76	30.3	78.3	18.1				
$Cs_{0.25}FA_{0.75}Sn_{0.5}Pb_{0.5}I_3$	1.25	0.798	31.1	78.4	19.1	4000 h, 80%	–	–	[S9]
$Cs_{0.15}FA_{0.85}Sn_{0.5}Pb_{0.5}I_3$	1.27	0.76	31.3	73	17.4	–	–	300 h, air, 65%	[S10]
$Cs_{0.3}FA_{0.7}Sn_{0.3}Pb_{0.7}I_3$	1.34	0.787	29.1	79.9	18.3	–	750 h, 80%	–	[S11]
GDR-Pb^0 (8.5)	1.26	0.84	30.37	72.24	18.34	–	700 h, 80%	2352 h, N_2, 81%	[S12]
GDR-Pb^0 (18.7)		0.86	31.55	73.64	20.01	–	–	–	
$Cs_{0.2}FA_{0.8}Pb_{0.5}Sn_{0.5}I_3$	1.24	0.86	31.5	77.9	21.10	–	–	–	[S13]

*MPP: continuous operation stability with maximum power point (MPP) tracking under 1-sun illumination.

Table S2 Peak parameters and assignments of Pb 4f and Sn 3d XPS signals for perovskites prepared with or without additive doping

Elements	Binding Energy (eV)	Sample	Affiliation
Pb	137.98/142.88[a)]	Control	**Pb** in PbO[S14–S18], Pb_3O_4[S19, S20], Pb[S20], and PbS[S21]
	137.78/142.68[a)]	D-HLH	**Pb** in PbO[S22–S27], and PbS[S21, S22, S28]
Sn	487.28/495.68[b)]	Control	**Sn** in SnO_2[S29–S31], $SnO_{1.65}$[S31], $SnCl_2$[S32], $SnF(C_6H_5)_3$[S33], $Sn(C_6H_5)_2Cl_2$[S34], $SnCl_4(C_5H_5N)_2$[S35], $SnCl_3(C_2H_5)(C_5H_5N)_2$[S35], and $SnCl_3(C_6H_5)(C_5H_5N)_2$[S35]
	487.08/495.48[b)]	D-HLH	**Sn** in SnO[S20, S36], SnF_2[S20, S37], SnO_2[S30, S31, S38–S40], $SnO_{1.65}$[S31], $SnF_2(CH_3)_2$[S35], $Sn(CH_3)_2SO_4$[S35], $SnCl(C_6H_5)_3$[S20], $Sn(C_6H_5)_4$[S35], $SnCl_2(CH_3)_2(SO(CH_3)_2)_2$[S35], and $Sn(C_6H_5)_3(C_9H_6NO)$[S41]

a) Pb $4f_{7/2}/4f_{5/2}$; b) Sn $3d_{5/2}/3d_{3/2}$

Table S3 Peak parameters and assignments of O 1s XPS signals for perovskites prepared with and without additive doping

Sample	O in inorganic molecule			O in organic molecule		
	Binding energy (eV)	FWHM [c)] (eV)	Atomic ratio (%)	Binding energy (eV)	FWHM [c)] (eV)	Atomic ratio (%)
Control	530.96[S40, S42]	1.63	46	532.21[S43, S44]	1.90	54
D-HLH	530.62[S45, S46]	1.60	27	531.82[S47, S48, S49–S62]	2.19	73

c) Full width at half maximum.

Table S4 FTIR spectral data for perovskites prepared with and without additive doping

Sample	Wavenumber (cm^{-1})		
	N–H stretching		C=O stretching
Control	3411	3270	1633
D-HLH	3401	3231	1607

Table S5 Photovoltaic parameters of PSCs prepared using different concentrations of D-HLH

D-HLH concentration (mg mL^{-1})	V_{oc} (V)	J_{sc} (mA cm^{-2})	FF (%)	PCE (%)
5	0.81	29.10	69.89	16.47
10	0.88	30.56	80.36	21.61
20	0.72	28.93	64.04	13.34

Table S6 Photovoltaic parameters of champion PSCs prepared with and without D-HLH

Device	Scan	V_{oc} (V)	J_{sc} (mA cm^{-2})	FF (%)	PCE (%)	Integrated J_{sc} (mA cm^{-2})
Control	Reverse	0.77	27.26	69.13	14.51	27.12
	Forward	0.76	27.41	67.54	14.07	
D-HLH	Reverse	0.88	30.56	80.36	21.61	29.86
	Forward	0.88	30.55	78.26	21.04	

Table S7 Photovoltaic parameters of champion tandem devices prepared with D-HLH

Device	Scan	V_{oc} (V)	J_{sc} (mA cm^{-2})	FF (%)	PCE (%)
Front cell	Reverse	1.18	16.21	82.15	15.71
	Forward	1.18	16.19	81.61	15.59
Back cell	Reverse	0.88	30.56	80.36	21.61
	Forward	0.88	30.55	78.26	21.04
Tandem-D-HLH	Reverse	2.03	14.42	81.37	23.82
	Forward	2.03	14.32	81.12	23.58

Table S8 Fitting parameters for TRPL curves of perovskite films

Sample	τ_{avg} (ns)	τ_1 (ns)	τ_2 (ns)	A_1 (%)	A_2 (%)
Control	9.14	1.77	13.44	81.58	18.42
D-HLH	28.81	3.39	31.93	53.67	46.33

$F(t) = A_1\exp(-t/\tau_1) + A_2\exp(-t/\tau_2) + y_0$

where τ_1 and τ_2 are the fast and slow decay times, respectively, and A_1 and A_2 are coefficients.

Table S9 Related parameters fitted from the equivalent circuit for EIS spectral measurement

Device	R_s (Ω)	R_{ct} (Ω)	C (nF)	R_{rec} (Ω)	CPE (nF)
Control	60.11	20,210	15	14,560	3970
D-HLH	42.06	3384	7783	19,220	28.21

Supplementary References

[S1] G.E. Eperon, T. Leijtens, K.A. Bush, R. Prasanna, T. Green et al., Perovskite-perovskite tandem photovoltaics with optimized band gaps. Science **354**(6314), 861–865 (2021). https://doi.org/10.1126/science.aaf9717

[S2] R. Prasanna, A. Gold-Parker, T. Leijtens, B. Conings, A. Babayigit et al., Band gap tuning via lattice contraction and octahedral tilting in perovskite materials for photovoltaics. J. Am. Chem. Soc. **139**(32), 11117–11124 (2021). https://doi.org/10.1021/jacs.7b04981

[S3] Y. Zong, N. Wang, L. Zhang, M.G. Ju, X.C. Zeng et al., Homogenous alloys of formamidinium lead triiodide and cesium tin triiodide for efficient ideal-bandgap perovskite solar cells. Angew. Chem. Int. Ed. **56**(41), 12658–12662 (2021). https://doi.org/10.1002/anie.201705965

[S4] T. Leijtens, R. Prasanna, K.A. Bush, G.E. Eperon, J.A. Raiford et al., Tin–lead halide perovskites with improved thermal and air stability for efficient all-perovskite tandem solar cells. Sustain. Energy Fuels **2**(11), 2450–2459 (2021). https://doi.org/10.1039/C8SE00314A

[S5] Y. Zong, Z. Zhou, M. Chen, N.P. Padture, Y. Zhou, Lewis-adduct mediated grain-boundary functionalization for efficient ideal-bandgap perovskite solar cells with superior stability. Adv. Energy Mater. **8**(27), 1800997 (2021). https://doi.org/10.1002/AENM.201800997

[S6] R. Prasanna, T. Leijtens, S.P. Dunfield, J.A. Raiford, E.J. Wolf et al., Design of low bandgap tin–lead halide perovskite solar cells to achieve thermal, atmospheric and operational stability. Nat. Energy **4**(11), 939–947 (2021). https://doi.org/10.1038/s41560-019-0471-6

[S7] A.F. Palmstrom, G.E. Eperon, T. Leijtens, R. Prasanna, S.N. Habisreutinger et al., Enabling flexible all-perovskite tandem solar cells. Joule **3**(9), 2193–2204 (2019). https://doi.org/10.1016/j.joule.2019.05.009

[S8] M.T. Klug, R.L. Milot, R.L. Milot, J.B. Patel, T. Green et al., Metal composition influences optoelectronic quality in mixed-metal lead–tin triiodide perovskite solar absorbers. Energy Environ. Sci. **13**(6), 1776–1787 (2021). https://doi.org/10.1039/D0EE00132E

[S9] J. Werner, T. Moot, T.A. Gossett, I.E. Gould, A.F. Palmstrom et al., Improving low-bandgap tin-lead perovskite solar cells via contact engineering and gas quench processing. ACS Energy Lett. **5**(4), 1215–1223 (2021). https://doi.org/10.1021/acsenergylett.0c00255

[S10] H. Liu, J. Sun, H. Hu, Y. Li, B. Hu et al., Antioxidation and energy-level alignment for improving efficiency and stability of hole transport layer-free and methylammonium-free tin-lead perovskite solar cells. ACS Appl. Mater. Interfaces **13**(37), 45059–45067 (2021). https://doi.org/10.1021/acsami.1c12180

[S11] J. Tong, J. Gong, M. Hu, S.K. Yadavalli, Z. Dai et al., High-performance methylammonium-free ideal-band-gap perovskite solar cells. Matter **4**(4), 1365–1376 (2021). https://doi.org/10.1016/J.MATT.2021.01.003

[S12] W. Zhang, X. Li, S. Fu, X. Zhao, X. Feng et al., Lead-lean and MA-free perovskite solar cells with an efficiency over 20%. Joule **5**, 2904-1914 (2021). https://doi.org/10.1016/j.joule.2021.09.008

[S13] Z. Yu, X. Chen, S.P. Harvey, Z. Ni, B. Chen et al., Gradient doping in Sn–Pb perovskites by barium ions for efficient single-junction and tandem solar cells. Adv. Mater., (2022). https://doi.org/10.1002/adma.202110351

[S14] J.M. Baker, R.W. Johnson, R.A. Pollak, Surface analysis of rf plasma oxidized in and PbInAu films using esca. J. Vac. Sci. Technol. **16**(5), 1534–1541 (1979). https://doi.org/10.1116/1.570243

[S15] C. Hinnen, C.N. Huong, P. Marcus, A comparative X-ray photoemission study of $Bi_2Sr_2CaCu_2O_8^{+\delta}$ and $Bi_{1.6}Pb_{0.4}Sr_2CaCu_2O_8^{+\delta'}$. J. Electron Spectros. Relat. Phenomena **73**(3), 293–304 (1995). https://doi.org/10.1016/0368-2048(94)02288-7

[S16] H. Kanai, M. Yoshiki, M. Hayashi, R. Kuwae, Y. Yamashita, Grain-boundary-phase identification of a lead-based relaxor by X-ray photoelectron spectroscopy. J. Am. Ceram. Soc. **77**(8), 2229–2231 (1994). https://doi.org/10.1111/j.1151-2916.1994.tb07128.x

[S17] G. Gökagaç, B.J. Kennedy, Potential-dependent surface segregation in lead + ruthenium pyrochlore $Pb_2Ru_2O_{7-y}$. J. Electroanal. Chem. **353**(1–2), 71–80 (1993). https://doi.org/10.1016/0022-0728(93)80287-R

[S18] P.A. Bertrand, P.D. Fleischauer, X-Ray photoelectron spectroscopy study of the surface adsorption of lead naphthenate. J. Vac. Sci. Technol. **17**(6), 1309–1314 (1980). https://doi.org/10.1116/1.570661

[S19] C. Barriga, S. Maffi, L.P. Bicelli, C. Malitesta, Electrochemical lithiation of Pb_3O_4. J. Power Sources **34**(4), 353–367 (1991). https://doi.org/10.1016/0378-7753(91)80101-3

[S20] W.E. Morgan, J.R.V. Wazer, Binding energy shifts in the X-ray photoelectron spectra of a series of related group IVa compounds. J. Phys. Chem. **77**(7), 964–969 (1973). https://doi.org/10.1021/J100626A023

[S21] A.R.H.F. Ettema, C. Haas, An X-ray photoemission spectroscopy study of interlayer charge transfer in some misfit layer compounds. J. Phys. Condens. Matter **5**(23), 3817–3826 (1993). https://doi.org/10.1088/0953-8984/5/23/008

[S22] D.S. Zingg, D.M. Hercules, D.M. Hercules, Electron spectroscopy for chemical analysis studies of lead sulfide oxidation. J. Phys. Chem. **82**(18), 1992–1995 (1978). https://doi.org/10.1021/j100507a008

[S23] V.I. Nefedov, M.N. Firsov, I.S. Shaplygin, Electronic structures of $MRhO_2$, MRh_2O_4, $RhMO_4$ and Rh_2MO_6 on the basis of X-ray spectroscopy and ESCA data. J. Electron Spectros. Relat. Phenomena **26**(1), 65–78 (2021). https://doi.org/10.1016/0368-2048(82)87006-0

[S24] L.R. Pederson, Two-dimensional chemical-state plot for lead using XPS. J. Electron Spectros. Relat. Phenomena **28**(2), 203–209 (2021). https://doi.org/10.1016/0368-2048(82)85043-3

[S25] J.A. Taylor, D.L. Perry, An X-ray photoelectron and electron energy loss study of the oxidation of lead. J. Vac. Sci. Technol. A **2**(2), 771–774 (2021). https://doi.org/10.1116/1.572569

[S26] J.F. Moulder, W.F. Stickle, P.E. Sobol, K.D. Bomben, *Handbook of X-ray Photoelectron Spectroscopy: A Reference Book of Standard Spectra for Identification and Interpretation of XPS Data*. Physical Electronics Division, Perkin-Elmer Corporation, (1992).

[S27] O. Sakurada, M. Taga, H. Takahashi, X-ray photoelectron spectroscopic study of the stabilization of lead with a palladium modifier in graphite furnace aas. Bunseki Kagaku **38**(9), 407–412(2021). https://doi.org/10.2116/bunsekikagaku.38.9_407

[S28] K. Laajalehto, I. Kartio, P. Nowak, XPS study of clean metal sulfide surfaces. Appl. Surf. Sci. **81**(1), 11–15 (1994). https://doi.org/10.1016/0169-4332(94)90080-9

[S29] Ş. Süzer, T. Voscoboinikov, K.R. Hallam, G.C. Allen, Electron spectroscopic investigation of Sn coatings on glasses. Fresenius J. Anal. Chem. **355**(5), 654–656 (1996). https://doi.org/10.1007/S0021663550654

[S30] M.A. Stranick, A. Moskwa, SnO_2 by XPS. Surf. Sci. Spectra **2**(1), 50–54 (1993). https://doi.org/10.1116/1.1247724

[S31] W. Choi, H. Jung, S. Koh, Chemical shifts and optical properties of tin oxide films grown by a reactive ion assisted deposition. J. Vac. Sci. Technol. **14**(2), 359–366 (1996). https://doi.org/10.1116/1.579901

[S32] G.T. Baronetti, S.R. Miguel, O.A. Scelza, A.A. Castro, State of metallic phase in $PtSn/Al_2O_3$ catalysts prepared by different deposition techniques. Appl. Catal. **24**(1–2), 109–116 (1986). https://doi.org/10.1016/S0166-9834(00)81261-0

[S33] S. Hoste, D.F. Vondel, G.P. Kelen, XPS Spectra of organometallic phenyl compounds of P, As, Sb and Bi. J. Electron Spectros. Relat. Phenomena **17**(3), 191–195 (1979). https://doi.org/10.1016/0368-2048(79)85040-9

[S34] M. Andersson, J. Blomquist, B. Folkesson, R. Larsson, P. Sundberg, Esca, mössbauer and infrared spectroscopic investigations of a series of tin complexes. J. Electron Spectros. Relat. Phenomena **40**(4), 385–396 (1986). https://doi.org/10.1016/0368-2048(86)80047-0

[S35] H. Willemen, D.F. Vondel, G.P. Kelen, An ESCA study of tin compounds. Inorg. Chim. Acta **34**, 175–180 (1979). https://doi.org/10.1016/S0020-1693(00)94698-X

[S36] J.C.C. Fan, J.B. Goodenough, X-ray photoemission spectroscopy studies of Sn-doped indium-oxide films. J. Appl. Phys. **48**(8), 3524–3531 (1977). https://doi.org/10.1063/1.324149

[S37] P. Owens, P.A. Grutsch, V. Zeller, T.P. Fehlner, K. Siegbahn et al., Photoelectron spectroscopy of tin compounds. Acta Crystallogr. Sect. B **12**(6), 1431–1433 (1973). https://doi.org/10.1021/ic50124a045

[S38] S. Badrinarayanan, A.B. Mandale, V.G. Gunjikar, A.P.B. Sinha, Mechanism of high-temperature oxidation of tin selenide. J. Mater. Sci. **21**(9), 3333–3338 (1986). https://doi.org/10.1007/BF00553376

[S39] E. Çetinörgü, S. Goldsmith, Y. Rosenberg, R.L. Boxman, Influence of annealing on the physical properties of filtered vacuum arc deposited tin oxide thin films. J. Non. Cryst. Solids **353**(26), 2595–2602 (1996). https://doi.org/10.1016/j.jnoncrysol.2007.04.031

[S40] M.D. Giulio, G. Micocci, A. Serra, A. Tepore, R. Rella et al., SnO_2 thin films for gas sensor prepared by r.f. reactive sputtering. Sens. Actuat. B Chem. **25**(1–3), 465–468 (1995). https://doi.org/10.1016/0925-4005(94)01397-7

[S41] P. Umapathy, S. Badrinarayanan, A.P.B. Sinha, An ESCA study of tin (IV) and tin (II) chelates with substituted 8-quinolinols. J. Electron Spectros. Relat. Phenomena **28**(3), 261–266 (1983). https://doi.org/10.1016/0368-2048(83)80004-8

[S42] M.D. Giulio, A. Serra, A. Tepore, R. Rella, P. Siciliano et al., Influence of the deposition parameters on the physical properties of tin oxide thin films. Mater. Sci. Forum **203**, 143–148 (1996). https://doi.org/10.4028/www.scientific.net/msf.203.143

[S43] C.M. Barnes, B.J. Kennedy, An X-ray photoelectron spectroscopic study of arene chromium tricarbonyl complexes at 170K. J. Mol. Struct. **344**(3), 233–240 (1995). https://doi.org/10.1016/0022-2860(95)08461-4

[S44] G. Beamson, D. Briggs, High Resolution XPS of organic polymers: the scienta ESCA300 database. J. Chem. Educ. **70**(1), A25 (1993). https://doi.org/10.1021/ed070pa25.5

[S45] P.R. Moses, H.O. Finklea, J.R. Lenhard, R.W. Murray, L.M. Wier et al., X-ray photoelectron spectroscopy of alkylamine-silanes bound to metal oxide electrodes. Anal. Chem. **50**(4), 576–585 (1978). https://doi.org/10.1021/ac50026a010

[S46] W. Choi, H. Jung, S. Koh, Chemical shifts and optical properties of tin oxide films grown by a reactive ion assisted deposition. J. Vac. Sci. Technol. **14**(2), 359–366 (1996). https://doi.org/10.1116/1.579901

[S47] H. Binder, D. Sellmann, Röntgen-photoelektronenspektroskopische untersuchungen an pentacarbonyl- chrom-und -wolfram-komplexen mit stickstoffliganden / X-ray photoelectron studies of pentacarbonyl chromium and tungsten complexes with nitrogen ligands. Zeitschrift Für Naturforsch. B **33**(2), 173–179 (1978). https://doi.org/10.1515/ZNB-1978-0211

[S48] S. Srivastava, S. Badrinarayanan, A.J. Mukhedkar, X-ray photoelectron spectra of metal complexes of substituted 2,4-pentanediones. Polyhedron **4**(3), 409–414 (1985). https://doi.org/10.1016/S0277-5387(00)87000-X

[S49] C.D. Wagner, D.A. Zatko, R.H. Raymond, Use of the oxygen KLL auger lines in identification of surface chemical states by electron spectroscopy for chemical analysis. Anal. Chem. **52**(9), 1445–1451 (1980). https://doi.org/10.1021/AC50059A017

[S50] C.A. Tolman, W.M. Riggs, W.J. Linn, C.M. King, R.C. Wendt, Electron spectroscopy for chemical analysis of nickel compounds. Inorg. Chem. **12**(12), 2770–2778 (1973). https://doi.org/10.1021/IC50130A006

[S51] S. Lars, T. Andersson, M.S. Scurrell, Infrared and ESCA studies of a heterogenized rhodium carbonylation catalyst. J. Catal. **59**(3), 340–356 (1979). https://doi.org/10.1016/S0021-9517(79)80003-2

[S52] J. Peeling, F.E. Hruska, D.M. McKinnon, M.S. Chauhan, N.S. McIntyre, ESCA studies of the uracil base. The effect of methylation, thionation, and ionization on charge distribution. Can. J. Chem. **56** (18) , 2405–2411 (1978). https://doi.org/10.1139/v78-393

[S53] M.C. Burrell, Y.S. Liu, H.S. Cole, An X-ray photoelectron spectroscopy study of poly(methylmethacrylate) and poly(α-methylstyrene) surfaces irradiated by excimer lasers. J. Vac. Sci. Technol. **4**(6), 2459–2462 (1986). https://doi.org/10.1116/1.574091

[S54] G.P. López, D.G. Castner, B.D. Ratner, XPS O 1s binding energies for polymers containing hydroxyl, ether, ketone and ester groups. Surf. Interface Anal. **17**(5), 267–272 (1991). https://doi.org/10.1002/sia.740170508

[S55] D.G. Castner, B.D. Ratner, Surface characterization of butyl methacrylate polymers by XPS and static SIMS. Surf. Interface Anal. **15**(8), 479–486 (1990). https://doi.org/10.1002/SIA.740150807

[S56] J. Szépvölgyi, A. Tüdös, I. Bertóti, X-ray photoelectron spectroscopy studies on solid xanthates. J. Electron Spectros. Relat. Phenomena **50**(2), 239–250 (1990). https://doi.org/10.1016/0368-2048(90)87068-Y

[S57] J. Russat, Characterization of polyamic acid/polyimide films in the nanometric thickness range from spin-deposited polyamic acid. Surf. Interface Anal. **11**(8), 414–420 (1988). https://doi.org/10.1002/sia.740110803

[S58] T. Sugama, L.E. Kukacka, N. Carciello, N.J. Hocker, Study of interactions at water-soluble polymer/$Ca(OH)_2$ or gibbsite interfaces by XPS. Cem. Concr. Res. **19**(6), 857–867 (1989). https://doi.org/10.1016/0008-8846(89)90098-7

[S59] L.C. Lopez, D.W. Dwight, M.B. Polk, The $\pi \rightarrow \pi^*$ shake-up phenomena in polyesters containing backbone aromatic groups. Surf. Interface Anal. **9**(1–6), 405–409 (1986).

[S60] J. Gardella, S.A. Ferguson, R.L. Chin, $\pi^* \leftarrow \pi$ shakeup satellites for the analysis of structure and bonding in aromatic polymers by X-ray photoelectron spectroscopy. Appl. Spectrosc. **40**(2), 224–232 (1986). https://doi.org/10.1366/0003702864509565

[S61] G.C. Allen, I.S. Butler, C. Kirby, Characterization of ferrocene and (η^6-benzene) tricarbonylchromium complexes by X-ray photoelectron spectroscopy. Inorg. Chim. Acta **134**(2), 289–292 (1987). https://doi.org/10.1016/S0020-1693(00)88098-6

[S62] K. Prabhakaran, C.N.R. Rao, Adsorption of carbonyl compounds on clean and modified Cu(110) surfaces: a combined eels-ups study. Appl. Surf. Sci. **44**(3), 205–210 (1990). https://doi.org/10.1016/0169-4332(90)90051-Z
